# Effectiveness of molecular fingerprints for exploring the chemical space of natural products

**DOI:** 10.1186/s13321-024-00830-3

**Published:** 2024-03-25

**Authors:** Davide Boldini, Davide Ballabio, Viviana Consonni, Roberto Todeschini, Francesca Grisoni, Stephan A. Sieber

**Affiliations:** 1 TUM School of Natural Sciences, Department of Bioscience, Technical University of Munich, Center for Functional Protein Assemblies (CPA), 85748 Garching bei München, Germany; 2 Milano Chemometrics and QSAR Research Group, Department of Earth and Environmental Sciences, University of Milano-Bicocca, P.zza Della Scienza, 1, 20126 Milan, Italy; 3 Institute for Complex Molecular Systems and Department of Biomedical Engineering, Eindhoven University of Technology, Eindhoven, Netherlands; 4 Centre for Living Technologies, Alliance TU/e, WUR, UU, UMC Utrecht, Utrecht, Netherlands

**Keywords:** Fingerprint, Natural products, Virtual screening, Similarity, Supervised classification

## Abstract

Natural products are a diverse class of compounds with promising biological properties, such as high potency and excellent selectivity. However, they have different structural motifs than typical drug-like compounds, *e.g.*, a wider range of molecular weight, multiple stereocenters and higher fraction of *sp*^*3*^-hybridized carbons. This makes the encoding of natural products via molecular fingerprints difficult, thus restricting their use in cheminformatics studies. To tackle this issue, we explored over 30 years of research to systematically evaluate which molecular fingerprint provides the best performance on the natural product chemical space. We considered 20 molecular fingerprints from four different sources, which we then benchmarked on over 100,000 unique natural products from the COCONUT (COlleCtion of Open Natural prodUcTs) and CMNPD (Comprehensive Marine Natural Products Database) databases. Our analysis focused on the correlation between different fingerprints and their classification performance on 12 bioactivity prediction datasets. Our results show that different encodings can provide fundamentally different views of the natural product chemical space, leading to substantial differences in pairwise similarity and performance. While Extended Connectivity Fingerprints are the de-facto option to encoding drug-like compounds, other fingerprints resulted to match or outperform them for bioactivity prediction of natural products. These results highlight the need to evaluate multiple fingerprinting algorithms for optimal performance and suggest new areas of research. Finally, we provide an open-source Python package for computing all molecular fingerprints considered in the study, as well as data and scripts necessary to reproduce the results, at https://github.com/dahvida/NP_Fingerprints.

**Supplementary Information:**

The online version contains supplementary material available at 10.1186/s13321-024-00830-3.

## Introduction

Natural products (NPs) are a source of inspiration for drug discovery due to their high potency and biological selectivity, which has translated in remarkable success in treating infectious diseases and cancer [1]. However, cheminformatic modeling of NPs has been limited because of their diversity from typical drug-like molecules (on which computational pipelines are usually developed), *e.g.*, in terms of their broader molecular weight distribution, multiple stereocenters, a higher fraction of *sp* [3]-hybridized carbons and extended ring systems [2, 3]. This issue is further compounded by a lack of biological annotations for NPs [4] and the widespread presence of activity cliffs due to their highly specialized biological functions [1].

One of the key steps of cheminformatics pipelines is how to encode structural information into ‘machine-readable’ formats for further processing. This can be achieved through the so-called molecular descriptors [5], which convert selected molecular features into one or more numbers via a pre-defined algorithm. Among various descriptors applied to natural products [6, 7], molecular fingerprints—which convert a molecular structure into a vector—bear promise to capture structural information on natural products (*e.g.*, presence or absence of certain substructures). In fact, fingerprints generally provide satisfactory performance for quantitative structure–activity relationship (QSAR) modeling [8–10], even in the presence of activity cliffs [11]. Given the relevance of fingerprints in cheminformatics, over 30 years of research in the field have led to a broad and diverse selection of fingerprinting algorithms [12, 13]. However, while extensive research exists on the performance of these algorithms on synthetic, drug-like molecules, little is known about the best practices for natural products encoding.

Stemming from these observations, the aim of this study is to comprehensively compare and evaluate how different types of molecular fingerprints perform for modeling the NP chemical space, and ultimately to (a) provide effective recommendations to cheminformatics practitioners in the field of NPs, and (b) underscore future directions for the development of molecular fingerprints. We systematically compared 20 different molecular fingerprinting algorithms from four packages [14–18], on two cheminformatics tasks. First, we evaluate the similarity of fingerprints encoding using the COCONUT database [4], containing over 400,000 unique NPs from 52 different sources, and a wide variety of organisms, geographic locations and applications. Then, we evaluated the selected fingerprints for quantitative structure–activity relationship (QSAR) modeling, using 12 datasets from the CMNPD database. [19]

The diverse fingerprint behavior in similarity searches and QSAR modelling using NPs allowed us to shed on their effect in representing the chemical space of natural products.

## Materials and methods

### Dataset curation

#### Unsupervised analysis

We used the COCONUT database [4], which contains over 400,000 unique NPs from 52 different sources, including compounds from a wide variety of organisms, geographic locations and applications. We considered those natural products whose source organism was reported, as done in a previous study [20]. Solvent exclusion, salt removal and charge neutralization were performed with the ChEMBL structure curation package [21]. Compounds that failed this standardization step or have SMILES could not be parsed with RDKIT were removed. The resulting dataset included 129,869 unique natural products (Table 1), divided into six sources: plant, fungi, bacteria, marine, animal and mixed (defined for cases where the same natural product is produced by multiple organisms). Additional file 1: Table S1 details how many compounds were removed at each preprocessing step. Each class was characterized by a different diversity in terms of percentage of atomic scaffolds, which was computed by dividing the number of unique Bemis Murcko [22] scaffolds by the total number of compounds in each class (Table 1).

**Table 1:** Summary of the data used in this study, collected and curated from COCONUT

Class	Number of compounds	Dataset %	Number of scaffolds	Scaffold diversity %
Plant	87,135	67.1	21,546	24.7
Fungi	15,516	11.9	4905	31.6
Bacteria	12,338	9.5	3824	31.0
Marine	8876	6.8	2443	27.5
Mixed	5290	4.1	1744	33.0
Animal	714	0.5	366	51.3
All	129,869	100	31,567	24.3

The distribution into classes (NP sources) is strongly skewed towards the plant class, encompassing 67.1% of total compounds, followed by fungi, bacteria, marine, mixed and animal (0.5%). In terms of compound diversity, there are four compounds per scaffold on average. The only outlier in this regard is the animal class, which has a much higher scaffold diversity rate (51.3%). This behavior might be related to the low number of NPs annotated for this class, or to the presence of acyclic natural products (e.g. linear peptides), making the Murcko scaffolds not as informative.

To compare the chemical space of NPs to typical drug-like compounds, we also included the Drug Repurposing Hub library in our analysis [23]. We preprocessed this dataset following the same procedure as for COCONUT, yielding 6776 unique drugs.

### QSAR modeling

Concerning the supervised classification datasets, we standardized the natural products from the CMNPD database (Comprehensive Marine Natural Products Database) [19] as described above. We considered 12 different molecular property prediction tasks. To construct each task, we selected all NPs annotated with the desired property as the positive class and a random sample of NPs from CMNPD as the negative class, enforcing a minimum dataset size of 1000 compounds (Table 2).

**Table 2:** Summary of the classification datasets used in this study, collected and curated from CMNPD

Dataset	Number of compounds	Active compounds
Antibiotic	1000	112
Antiviral	1000	106
Antitumoral	1000	154
Antimalarial	1000	92
Antileishmanial	1000	20
Kinase C inhibition	1000	22
Serine Protease inhibition	1000	29
ATPase inhibition	1000	78
HIV	1000	178
Antifungal	1000	364
Anti-inflammatory	1000	156
Phosphatase inhibition	1000	95

Similar dataset generation procedures have been popularized for evaluating ligand-based virtual screening approaches [24–26], but they have the drawback of potentially introducing noise in the labels of the inactive compounds, since the negative class is constructed by sampling unlabeled molecules. However, this was necessary for our benchmark due to the scarcity of biological annotations for NPs, making it difficult to generate classification datasets where negative data had also been measured [3, 27].

### Molecular fingerprints

In total, we analyzed 20 different fingerprinting algorithms belonging to five different categories (Table 3). We used the default calculation parameters provided by the source package for each fingerprint.

**Table 3:** List of molecular fingerprints evaluated in this study, detailing for each the original publication year, the algorithm category, bit information type, number of bits, source package and parameters used for the calculation

Name	Year	Category	Type	Size	Source	Parameters
Topological Torsion (TT) [28]	1987	Path	Count	4096	RDKIT [14]	targetSize = 4
Atom Pair (AP) [29]	1985	Path	Count	4096	RDKIT [14]	N.A
Avalon [30]	2006	Path	Count	1024	RDKIT [14]	N.A
Daylight [31]	1973	Path	Binary	1024	CDK [15]	Depth = 7
Depth First Search (DFS) [32]	2005	Path	Binary	4096	jCompoundMapper [16]	Depth = 7
All Shortest Paths (ASP) [16]	2011	Path	Binary	4096	jCompoundMapper [16]	Depth = 7
RDKIT [14]	2012	Path	Binary	2048	RDKIT [14]	Depth = 7
Pharmacophore Pairs (PH2) [33]	2006	Pharmacophore	Binary	4096	jCompoundMapper [16]	N.A
Pharmacophore Triplets (PH3) [33]	2006	Pharmacophore	Binary	4096	jCompoundMapper [16]	N.A
MACCS [34]	2002	Substructure	Binary	166	RDKIT [14]	N.A
PubChem [35]	2009	Substructure	Binary	881	CDK [15]	N.A
ESTATE [36]	1995	Substructure	Binary	79	CDK [15]	N.A
Klekota-Roth (KR) [37]	2008	Substructure	Binary	4860	CDK [15]	N.A
Extended Connectivity (ECFP) [38]	2010	Circular	Binary	1024	RDKIT [14]	Radius = 2
Functional Class (FCFP) [38]	2010	Circular	Binary	1024	RDKIT [14]	Radius = 2
RAD2D [39]	2004	Circular	Binary	4096	jCompoundMapper [16]	N.A
LSTAR [16]	2011	Circular	Binary	4096	jCompoundMapper [16]	N.A
LINGO [40]	2005	String	Binary	1024	CDK [15]	N.A
MinHashed (MHFP) [18]	2018	String	Categorical	1024	Ref. [19]	Radius = 3
MinHashed Atom Pair (MAP4)^17^	2020	String	Categorical	1024	Ref. [18]	Radius = 2

Five categories of fingerprints were considered, based on the type of molecular information they capture:

- *Path-based fingerprints* generate molecular features by analyzing the paths through the molecular graph given a pair of atoms and hashing them inside a fixed-size vector [16]. For example, Depth First Search (DFS) represents a compound by storing all unique paths in its graph, obtained by using each atom as the path starting point and moving away up to a number of bonds *d*. [32] Another example of this class of algorithms are Atom Pair fingerprints (AP), where a molecule is described by collecting all possible triplets of two atoms and the shortest path connecting them [29].
- *Pharmacopohore fingerprints*, which are a variation of path-based fingerprints, where atoms are described by whether they are a pharmacophore point (e.g. whether they are hydrogen bond donors or acceptors) [33]. This leads to bit vectors that are less related to the compound structure, but instead try to encode how the molecule interacts with its chemical environment. Examples of this class of algorithms are Pharmacophore Pairs (PH2) and Pharmacophore Triplets (PH3) [33].
- *Substructure-based fingerprints,* in which each bit encodes whether the compound contains a predefined structural moiety [34, 37]. Examples of this class of algorithms are the MACCS structural keys and the PUBCHEM fingerprints [34, 35].
- *Circular fingerprints* also break up a target compound into different fragments like substructure-based fingerprints, but instead of relying on expert-defined structural patterns, they construct them dynamically from the molecular graph for each compound [38, 39]. To do so, they initially represent each atom according to some properties, such as atomic mass or valence. Then, for each atom, the numerical identifier of neighboring atoms is added, thus generating a fragment identifier. This process can be repeated several times, progressively increasing the radius of the neighborhood to consider when aggregating information. Finally, all unique fragments for a given molecule are hashed into a fixed-size vector. Typically, the difference between fingerprints belonging to this class lies in using different properties for the atom identifiers. For example, Extended Connectivity fingerprints (ECFP) use features such as the atomic number, atomic charge and so forth, while Functional Class fingerprints (FCFP) consider whether the atom is basic, acid, a hydrogen bond donor/acceptor etc [38].
- *String-based fingerprints* generate molecular representations by operating on the SMILES string of the compound, instead of its graph representation [18, 40]. For example, for a given dataset, LINGO fingerprints fragment the SMILES strings in fixed-size substrings and compute the total number of unique substrings across all compounds [40]. Then, each compound is encoded according to which SMILES substrings in the set it contains, using either counts or binary values. Another example of string-based algorithms are the MinHashed fingerprints (MHFP) [18]. This method works similarly to circular fingerprints, but instead of using atom identifiers, it considers the SMILES substring of a given fragment as its identifier. Each fragment identifier is then stored in a fixed-size vector via MinHash. MinHashed Atom Pair fingerprints (MAP4) [17] work similarly, but also consider the topological distance between atom pairs in the fragment for generating the fragment identifier.

Molecular fingerprints can be further characterized according to the information they encode in each element of the vector: binary fingerprints indicate the presence or absence of a given molecular pattern, count-based fingerprints have integer values specifying the number of occurrences of a given fragment and categorical fingerprints use numerical identifiers to describe the chemical motifs in the compound. [15–18]

### Similarity metrics

We used the Jaccard-Tanimoto similarity [41] to assess pairwise similarities between compounds for all fingerprints. For categorical fingerprints (MAP4 and MHFP), we used a modified version of the Jaccard-Tanimoto similarity which considers two bits as a match if they contain exactly the same integer, as introduced in a previous study [17, 18, 20]. To ensure comparability, count-based fingerprints were converted into binary bits, by only encoding whether a fragment is present or absent, and then pairwise similarities were measured as for the other encodings. This ensures that any variation in pairwise similarities between two fingerprint types is exclusively related to differences in how the vectors are computed, and not due to using different metrics.

### Pairwise distribution correlation analysis

For each type of fingerprint, evaluating all pairwise similarities on all compounds from the preprocessed version of the COCONUT dataset would be computationally infeasible, given that this would require calculating more than 8 billion similarity values. To mitigate this, we adopted a repeated resampling procedure which considered batches of 10,000 randomly selected NPs to compute the similarity, as:

- Given a sample of $n=\mathrm{10,000}$ compounds, we computed their fingerprints according to the 20 considered algorithms (Table 1), and for each type of fingerprint all the corresponding pairwise similarities.
- We concatenated the pairwise similarities in a matrix $\mathbf{B}(m\times p)$, with $m=\frac{10000*9999}{2}=49995000$ and $p = 20$, and calculated mean, standard deviation, median and percentiles of the distribution of the compound pairwise similarities for each type of fingerprint.
- Then, we computed the correlation matrix of $\mathbf{B}$, yielding a matrix $\mathbf{C}(20\times 20)$, which describes how well each fingerprint correlates with one another in terms of pairwise similarities for a given natural product batch.
- Finally, once all batches were processed, we averaged all statistics across all 50 iterations.

The same procedure was repeated for the Drug Repurposing Hub dataset, but since it only has 6776 unique compounds, the procedure was carried out without the use of batches.

### Unsupervised embeddings

We computed Uniform Manifold Approximation and Projection (UMAP) [42] embeddings for each fingerprint, using different metrics for each fingerprint numerical type as described in the Similarity metrics section. Each other parameter was set to its default value from the UMAP package [43]. We focused our analysis on the first batch of 10,000 molecules we used for the pairwise correlation analysis, since using the entire dataset would have been computationally infeasible. We verified that the class distribution and the chemical diversity for each batch is consistent with the values obtained for the whole dataset (Additional file 1: Tables S1-S2), ensuring that the UMAP analysis of the batch is representative of the entire chemical space we investigated.

### Classification

To assess how well each fingerprint can be used for QSAR modeling of natural products, we evaluated them on 12 different bioactivity prediction datasets. Each classification dataset (Table 2) was divided in three folds using an 80:10:10 ratio between training, validation and test set with scaffold split [44]. For each fingerprint type, we then trained two models:

- *Random Forest classifier (RF)* [45]*.* Bayesian hyperparameter optimization for 20 iterations, training on the training split and measuring the ROC-AUC on the validation set (hyperparameters: number of trees between 50 and 500 with a step of 50, maximum tree depth between 5 and 12 with a step of 2, the minimum number of samples per split between 2 and 20, minimum number of samples per leaf between 2 and 100, number of features as a choice between the logarithm, the square root or 10% of the fingerprint size). We finally trained on the training set and evaluated the performance on the test set with 5 replicates.
- *Dense Neural Network (DNN)* [46] with 2 hidden layers, batch normalization and dropout*.* Each DNN was trained for 100 epochs using AdamW as the optimizer and binary cross-entropy as the loss function on the training set. The parameters were optimized via Bayesian optimization for 20 iterations according to the ROC-AUC on the validation set. We tuned the number of units per layer (between 128 and 512 with a step of 128), the dropout rate (between 0 and 0.4), the learning rate (between 0.0001 and 0.05) and the batch size (between 16 and 64 with a step of 8). Once the optimal hyperparameters were determined on the validation set, we retrained on the training set and measured all metrics on the test set, repeating the procedure 5 times.

The classification performance was quantified using precision, recall, specificity, Matthews Correlation Coefficient (MCC), F1 score, balanced accuracy, ROC-AUC and PR-AUC [47]. Our selection ensures that our evaluation encompasses all aspects of a given classifier’s performance and is robust to class imbalance [48, 49]. To assess whether the any fingerprint was ranked differently than the others across all datasets, we first performed a Friedman test for each classification metric and classification model [50]. If the outcome of the Friedman test was statistically significant (α < 0.05), we then performed post-hoc tests (2-tailed Wilcoxon signed rank test with Benjamini–Hochberg correction, α < 0.05) to identify which fingerprint pair was significantly different [51, 52].

### Hardware and software

The analysis and calculation pipelines were implemented in Python 3.8, using JPype 1.4.1 to access packages originally written in Java. We used RDKIT 2022.9.5, CDK 2.2 and jCompoundMapper 1.0 for computing fingerprints, scipy 1.8.1 and numpy 1.22.3 for computing Tanimoto similarity and performing statistical tests, statsmodels 0.15 for adjust p-values with the Benjamini–Hochberg correction [53], RDKIT 2022.9.5 and chembl_structure_pipeline 1.2.0 for compound standardization, hyperopt 0.2.7 for Bayesian hyperparameter optimization [54], Pytorch 2.1.0 [55] for training the DNN models and scikit-learn 1.2.2 [56] for training the RF models and computing classification metrics. All calculations were carried out on a server with an AMD Ryzen Threadripper 3970 × 32-core CPU and 128GB of RAM, using all threads available. The code for reproducing the results, calculating all the considered fingerprints, along with the performance metrics for each individual dataset and classifier are provided for free in the following Github repository: https://github.com/dahvida/NP_Fingerprints.

## Results and discussion

### Pairwise similarity distribution

**Fig. 1:**
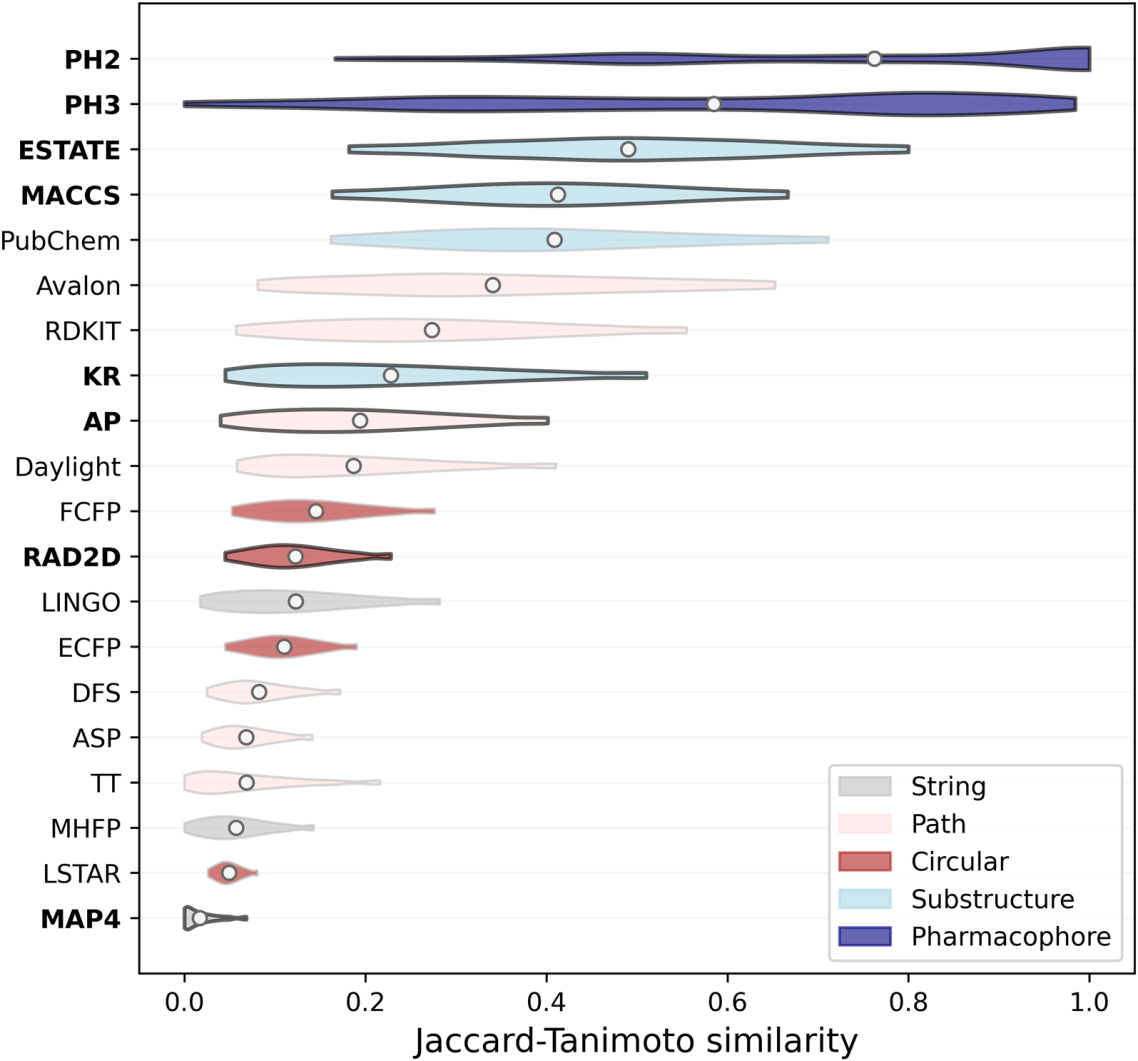
Jaccard-Tanimoto similarity distribution for each fingerprint across all possible pairwise comparisons in the natural product dataset. Violin plots indicate the percentiles of the distribution of Jaccard-Tanimoto similarities, with the circle indicating the median similarity value. The fingerprints where the similarity distribution on natural products is significantly different than the one obtained for drug-like compounds are highlighted in bold (Mann Whitney tests with Benjamini–Hochberg correction, α = 0.05)

We first analyzed the distribution of pairwise similarities across the COCONUT dataset (Fig. 1 and Table 4) and the Drug Repurposing Hub compounds (Additional file 1: Figure S1) to understand which fingerprints provide a more granular view for NPs and whether these patterns differ with drug-like molecules.

**Table 4:** Distribution statistics for the pairwise Jaccard-Tanimoto similarity scores obtained by each fingerprint across all batches of the COCONUT dataset

Fingerprint	Minimum	25th percentile	50th percentile	75th percentile	Maximum
MAP4	0.000	0.002	0.011	0.026	0.067
LSTAR	0.026	0.039	0.048	0.059	0.080
MHFP	0.000	0.028	0.052	0.082	0.141
TT	0.000	0.023	0.055	0.103	0.212
ASP	0.020	0.043	0.064	0.090	0.140
DFS	0.026	0.054	0.077	0.107	0.169
ECFP	0.046	0.082	0.108	0.137	0.190
LINGO	0.018	0.065	0.114	0.173	0.279
RAD2D	0.047	0.087	0.118	0.154	0.226
FCFP	0.053	0.099	0.139	0.186	0.275
Daylight	0.059	0.111	0.171	0.249	0.404
AP	0.042	0.113	0.184	0.267	0.399
KR	0.047	0.125	0.210	0.317	0.504
RDKIT	0.062	0.166	0.261	0.371	0.550
Avalon	0.084	0.211	0.326	0.467	0.648
PubChem	0.167	0.294	0.396	0.516	0.706
MACCS	0.168	0.313	0.410	0.511	0.667
ESTATE	0.186	0.364	0.500	0.615	0.799
PH3	0.036	0.322	0.638	0.830	0.952
PH2	0.228	0.500	0.875	1.000	1.000

On the COCONUT dataset, Pharmacological fingerprints (PH2 and PH3) have the broadest distribution of pairwise similarities as well as the highest median Jaccard-Tanimoto similarity. Crucially, both distributions consistently reach similarity scores above 0.95, especially for PH2, indicating that even though the dataset is without replicates, according to these embedding many compounds are nearly indistinguishable. This is consistent with how this class of fingerprint is computed: instead of capturing information pertaining to the molecular structure, these embeddings try to describe molecules in terms of how they interact with their biological environment through their pharmacophores. As such, compounds that have very different chemical structures can still have identical pharmacophoric points, which is reflected by their high similarity scores in terms of PH2 and PH3 fingerprints. This shows that these featurization approaches are well suited for scaffold hopping in the NP chemical space, but their inability to separate structurally different compounds might be problematic for other QSAR applications. On the Drug Repurposing Hub both fingerprints achieve significantly lower median Jaccard-Tanimoto similarities (Mann Whitney test with Benjamini–Hochberg correction, α = 0.05), especially PH3. This might be due to the smaller dataset size and higher scaffold diversity compared to COCONUT (62% instead of 24%), which generally lowers all median Jaccard-Tanimoto similarities for all fingerprints. Another factor could be a larger range of pharmacophoric arrangements between the drugs considered for the analysis, consistently with the broad range of therapeutic targets of the molecules of this library. In that case, this pattern would affect PH3 more since it considers triplets instead of pairs, which leads to a higher number of potential combinations.

Next, substructure-based fingerprints like MACCS, ESTATE, PubChem and KR tend to achieve the highest Jaccard-Tanimoto similarity scores. This is consistent with their reliance on predefined fragments, rather than processing each molecular graph individually. Since the fragments chosen by these fingerprints were defined for small molecules, only a fraction of them is usually found in NPs, while other highly informative NP-like substructures are not encoded. This reduces the average bit variance across the fingerprints, leading to more similar vectors overall. These types of embeddings can therefore be problematic for the NP chemical space, unless custom fragments are added to account for the molecular distribution shift and feature selection is used to remove uninformative bits. This issue seems especially pronounced for MACCS and KR, since they achieve significantly lower median similarity scores (Mann Whitney test with Benjamini–Hochberg correction, α = 0.05) on the Drug Repurposing Hub, shifting from 0.40 and 0.21 to 0.32 and 0.13. In contrast, PubChem and ESTATE remain comparable. This trend reflects the focus MACCS and KR have on drug discovery, thus biasing the fragment choice on relevant motifs for the drug-like chemical space. [34, 37]

Both path-based and circular fingerprints have median values of Jaccard-Tanimoto similarity around 0.1, and narrower score distributions. Two exceptions to this pattern are RDKIT, which has a comparable distribution to substructure-based encodings, and LSTAR, which has a very narrow distribution with a lower median similarity than other circular or path-based fingerprints. A similar trend is observed on the Drug Repurposing Hub, with path-based and circular fingerprints being distributed between 0.2 and 0.1 median Jaccard-Tanimoto similarity scores.

When it comes to MinHashed fingerprints, the low median Jaccard-Tanimoto scores obtained by MAP4 on both COCONUT and the Drug Repurposing Hub (less than 0.02) could be related to two factors. First, this fingerprint uses categorical encodings, which means that their similarity is computed via the modified Jaccard-Tanimoto similarity. According to that metric, for two bits to be considered a match it is not enough that they are both non-zero, but they must have the same integer value. As such, the fraction of matching bits given two fingerprints of this type tends to be much lower compared to binary fingerprints. Second, it could be that MinHashing paths rather than circular fragments lead to more potential categorical values for each bit, reducing the number of bit matches when comparing two fingerprints. This would explain why MHFP has higher median pairwise Jaccard-Tanimoto similarity.

To further analyze the distribution of pairwise similarity scores, we evaluated the average “bit saturation” [57] of each fingerprint on the COCONUT and Drug Repurposing Hub datasets (Additional file 1: Table S5). On average, most fingerprints have higher saturation scores for natural products than for synthetic drugs, indicating the presence of larger, and more complex molecular structures [1]. One exception to this trend is substructure fingerprints, which have lower bit saturation on natural products than drug-like compounds. This is caused by the presence of uninformative fragments for natural products in the fingerprint definition, leading to less bits being set when encoding a given compound.

### Fingerprint correlation analysis

**Fig. 2:**
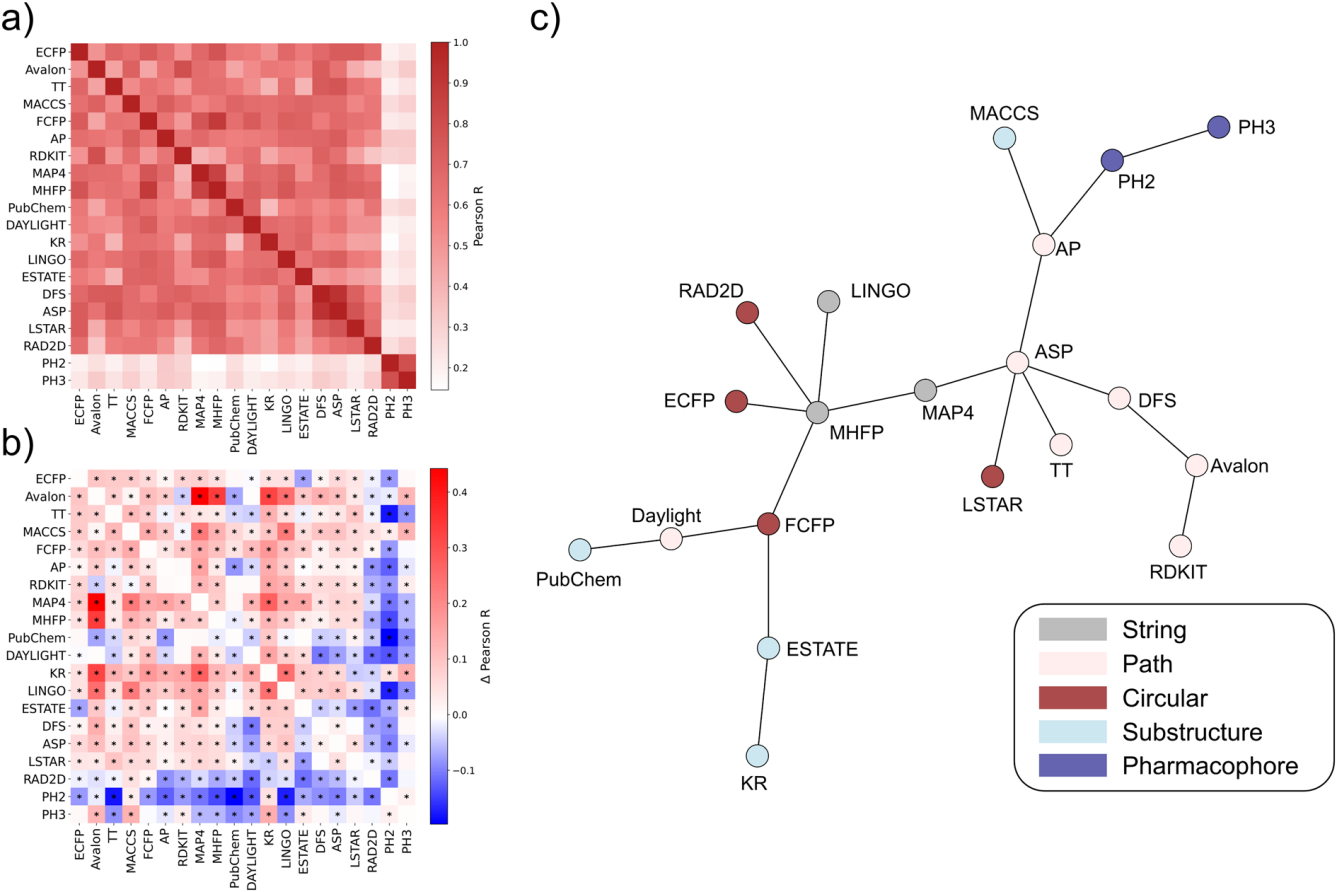
Jaccard-Tanimoto similarity correlation analysis for all fingerprints. **a** Correlation matrix for all fingerprints evaluated in this study on the COCONUT dataset. **b** Difference between the correlation matrix obtained for the COCONUT dataset and for the Drug Repurposing Hub. Positive values indicate higher fingerprint correlation in the NP space, while negative values denote higher correlation in the drug-like space. Asterisks denote statistical significance according to one-sample Mann Whitney tests with Benjamini–Hochberg correction (α = 0.05). **c** MST constructed from the fingerprint correlation matrix obtained for the NP chemical space. Each encoding is colored on the basis of its category

To better evaluate which fingerprints provide different views of the NP chemical space, we calculated the Pearson correlation coefficient between each pairwise similarity score across all fingerprints (Fig. 2a). It is immediately apparent that both pharmacological fingerprints (PH2 and PH3) are outliers, given that they are extremely correlated between each other and almost completely uncorrelated with all others. This could be related to the fact that, unlike the other fingerprints analyzed, these fingerprints describe the occurrence of ‘fuzzy’ pharmacophoric points, rather than focusing on the presence or occurrence of functional groups and substructures.

When evaluating the correlations between the other fingerprints, it becomes clear that some fingerprints are highly correlated (above 0.8) with each other. MAP4 and MHFP (string fingerprints), as well as DFS and ASP (pharmacophore fingerprints) show high Pearson correlation coefficients (0.85 and 0.92 respectively). This is consistent with the fact that they belong to the same class, and hence are based on a similar featurization strategy. The first pair is especially interesting, given that while they both rely on SMILES substrings, MAP4 relies on topological distances between atom pairs, while MHFP considers circular neighborhoods around atoms for its fragments. This difference is also consistent when looking at their correlation with other circular fingerprints, such as ECFP and FCFP: MHFP strongly correlates with both (0.77 and 0.88), while MAP4 to a lesser extent (0.67 and 0.77).

To quantitatively assess which fingerprint correlation pairs change the most when considering the NP chemical space specifically, we first computed the correlation matrix for the Drug Repurposing Hub dataset (Additional file 1: Figure S2) and then calculated the Pearson R difference between the values obtained for NPs and the ones for drugs (Fig. 2b). For most encoding pairs, the difference is statistically significant, as shown in Fig. 2b (one-sample Mann Whitney tests with Benjamini Hochberg correction, α = 0.05). Most fingerprints are more correlated in the NP space than in the drug-like space, with an average Pearson R difference of around 0.1, except for PH2 and PH3, which instead are less correlated to the others. The correlation increase for the majority of fingerprints likely reflects the fact that many bits are less informative for NPs than they are for drugs, thus reducing the ability of different fingerprints to capture molecular similarity from different perspectives. Notably, the correlation difference between Avalon and KR, MAP4 and MHFP is especially high (0.4), indicating that their chemical space mapping is very similar with NPs but not with drug-like compounds. On the other hand, the correlation decrease observed for PH2 and PH3 hints at the fact that similarities computed using these encodings tend to be outliers in the NP chemical space, as observed when evaluating their distribution and as discussed below when analyzing their unsupervised embeddings.

Another key difference between natural products and drug-like compounds is that the former tend to have a higher number of repetitive chemical moieties, which can be accurately captured by using count-based fingerprints. To evaluate how using counts affects the encoding of natural products, we repeated the Pearson correlation analysis for all count-based fingerprints (AP, TT and Avalon) for both COCONUT and Drug Repurposing Hub datasets (Additional file 1: Table S6). While there is a consistently high similarity score correlation between using counts and binary bits for a given fingerprint (e.g. AP has a Pearson R of 0.75 on the COCONUT dataset), there is a statistically significant difference for all fingerprints in how correlated counts and bits are when comparing natural products and drug-like compounds. Specifically, AP and Avalon show less correlation on natural products than on drug-like molecules, decreasing by 0.01 and 0.03 in terms of Pearson R respectively. In contrast, TT shows higher Pearson R on medicinal chemistry compounds. These results suggest therefore that count-based AP and Avalon fingerprints are more appropriate at capturing repetitive chemical moieties found in natural products, since there is larger disagreement between counts and binary fingerprints in terms of molecular similarity.

### Visualizing fingerprint similarity via minimum spanning tree

To further aid in the visualization of the similarities between fingerprints, we constructed a Minimum Spanning Tree (MST) [58] from the correlation matrix (Fig. 2c). The Minimum Spanning Tree was performed by calculating the Pearson correlation distance from the correlation matrix (Fig. 2a), as $\mathbf{P}=1-\mathbf{C}$, where $\mathbf{C}$ is the correlation matrix with all positive values.

Path-based encodings are in proximity of each other except for Daylight, which is linked to PubChem and FCFP, and RDKIT, which is only connected to Avalon. DFS is the fingerprint of this category that is most correlated within its category, reaching all other path-based algorithms in at most two steps within the MST. Circular and string-based fingerprints are mostly interconnected with each other, apart from LSTAR. MHFP connects with FCFP, ECFP and RAD2D, consistently with the fact that it also relies on circular fragments, while MAP4 connects with ASP, which likely reflects the fact that it encodes topological distances between atom pairs. FCFP is unique among all fingerprints, given that it connects with a fingerprint from all other categories except for pharmacophore-based encodings. This is especially surprising given that FCFP uses pharmacophoric information for the atom identifiers, which one might assume would lead to higher correlation with PH2 and PH3. Furthermore, it is notable that ECFP and FCFP correlate more strongly with MHFP than with each other, despite using the same algorithm except for the atom definitions. This seems to suggest that MinHashing SMILES substrings provide a hybrid representation that captures both chemical and pharmacophoric properties of the molecule. Substructure-based fingerprints are the most diverse, with only KR not connecting to algorithms belonging to different categories. PubChem and MACCS are linked to Daylight and AP respectively, while ESTATE is related to FCFP. This indicates that the fragment choices of these encodings are mostly orthogonal with each other and that, overall, this category is correlated to path-based and circular approaches. Pharmacophore fingerprints are separated from all other categories, consistently with the correlation matrix and their pairwise similarity distribution. The closest neighbor from a different class is AP, which is connected to PH2, reflecting the fact that that both algorithms rely on distances between atom pairs.

Finally, this analysis confirms the assumption that, when deciding which fingerprint to use for similarity searches or QSAR modeling, the optimal strategy is to consider approaches belonging to different categories in order to minimize redundancy.

### Similarity search ranking comparison

Similarity searching is often employed to identify the top K most similar compounds to a query molecule, e.g. to identify new bioactive molecules given a ligand for a protein of interest according to the similarity principle [59–61]. To examine whether different fingerprints would produce the same hits when used for similarity-based virtual screening, we repeated the sampling procedure described for the correlation comparison analysis and calculated for each compound the top 1% most similar molecules. We performed this procedure for each fingerprint and given a pair of encodings, we measured how many hits were ranked in the top 1% by both approaches. Finally, to evaluate whether natural products and drug-like compounds yield different results, we repeated this procedure for both the COCONUT and Drug Repurposing Hub datasets (Additional file 1: Figure S3).

Most fingerprint pairs exhibit an overlap score of approximately 25% on natural products, meaning that given a query molecule, 25% of the virtual screening hits are the same using both fingerprints. DFS and ASP show higher overlap than average (62%), consistently with the use of similar path enumeration algorithms to encode chemical graphs. When comparing the results obtained on COCONUT with the ones from Drug Repurposing Hub, the change in overlap percentage is between − 4% and 10% and is statistically significant for most fingerprint pairs (Additional file 1: Figure S3b). Finally, the ranking overlap difference is mostly consistent with the change observed in terms of similarity score correlation. For example, ESTATE and RAD2D fingerprints are generally more diverse from other encodings in the natural product space both in terms of top 1% ranking and overall pairwise Tanimoto correlation.

### Exploring the natural product chemical space via dimensionality reduction

**Fig. 3:**
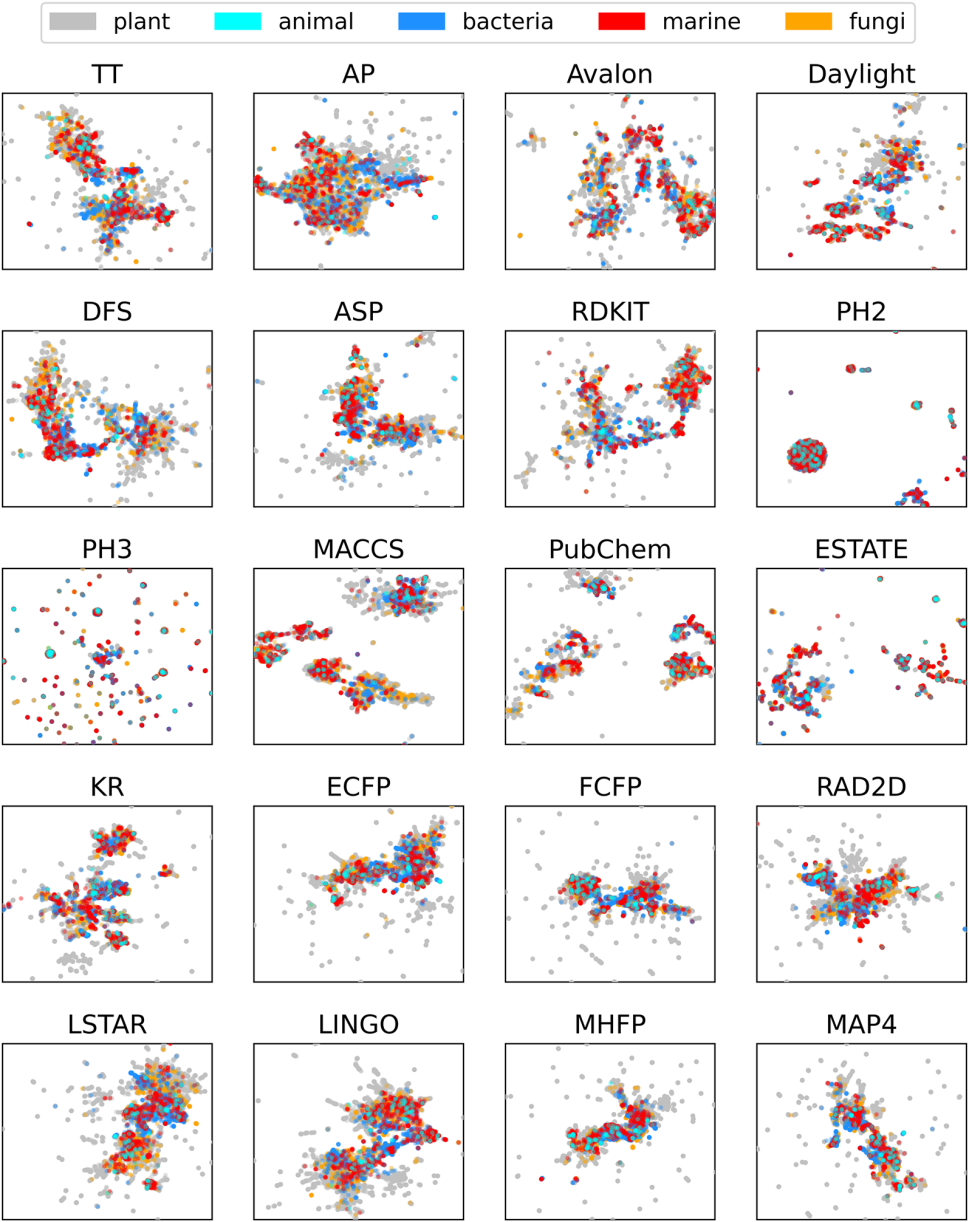
Plot of UMAP embeddings for each fingerprint. Chemicals are colored on the basis of their source organism

To analyze the effect that fingerprints have on capturing the distribution of NPs in the chemical space, we compared their bidimensional embeddings via UMAP (Fig. 3). Additionally, we investigated whether any embedding could separate NPs according to different taxonomical classes, given that different organisms produce biomolecules in different ranges of molecular weight, fraction of *sp3*-hybridized carbon and logP [20]. To do so, we colored the UMAP projections of NPs according to their taxonomy, after removing all compounds originating from multiple organisms.

Overall, no fingerprint can visually separate NPs according to their taxonomy, indicating that while different organism types generally produce compounds with different molecular properties, there is a significant overlap between these distributions. This is also consistent with the non-negligible fraction of NPs which are produced by multiple taxonomical classes found in COCONUT (4%).

Concerning the quality of the embeddings, PH2 and PH3 have atypical behaviors compared to all other fingerprints, with the former having one large compound group separated from everything else, while the latter showing none. These patterns are likely caused by the very broad similarity distribution observed for these fingerprints, making it difficult for the UMAP algorithm to preserve the manifold correctly.

Substructure-based fingerprints provide clear grouping of compounds according to their chemical structure, as shown by the clearly separated clusters in their embeddings, although this does not necessarily correlate with taxonomical information.

Path-based and circular fingerprints instead seem to provide much more uniform embeddings, causing most clusters to be closer together than for substructure-based approaches and making the manifold internal structure less distinct.

Finally, MAP4 and MHFP have comparable embeddings to path-based and circular fingerprints, albeit with a larger number of isolated compounds.

### Classification performance

Depending on the classifier, metric and assay of interest, different fingerprints perform the best, with no clear favorite across the board. The only consistent pattern across all analyses is that pharmacophore fingerprints tend to underperform for classification, likely due to their inability to precisely distinguish chemical motifs.

**Fig. 4:**
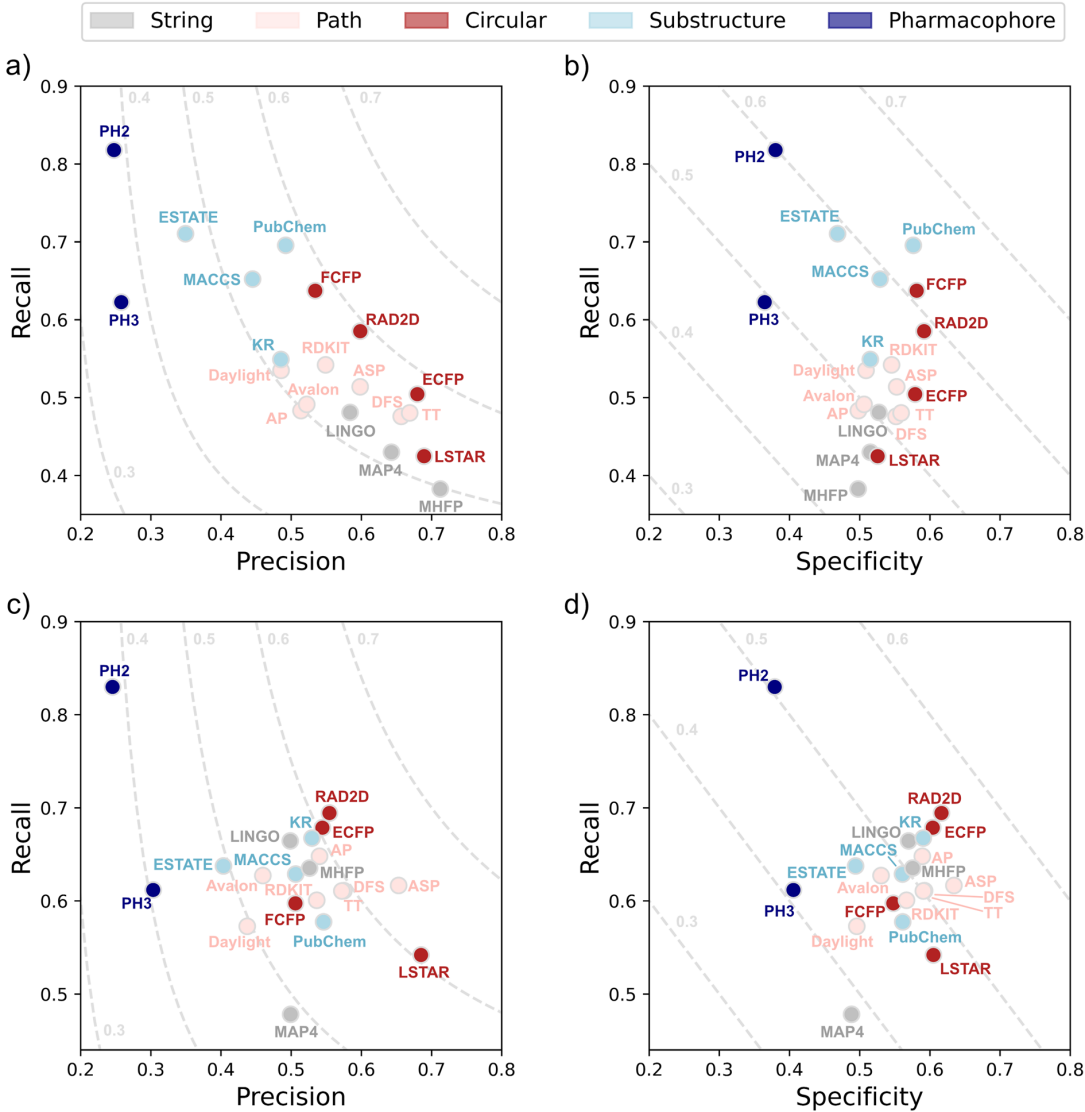
Mean classification performance of each fingerprint across all datasets. **a** Recall versus precision plot for Random Forest, contour lines denote F1 scores. **b** Recall versus specificity plot for Random Forest, contour lines indicate balanced accuracy. **c** Recall versus precision plot for Dense Neural Networks, contour lines denote F1 scores. **d** Recall versus specificity plot for Dense Neural Networks, contour lines indicate balanced accuracy

When considering RF, in terms of global classification metrics, on average RAD2D achieves the best MCC (0.506), LSTAR outperforms all alternatives in terms of ROC-AUC (0.900) and MHFP performs the best in terms of PR-AUC (0.669), as shown in Additional file 1: Table S7. ASP is also a competitive option, ranking first in terms of ROC-AUC on 3 datasets out of 12 (Additional file 1: Table S9). In terms of individual datasets, LSTAR is especially promising for antiviral activity prediction (0.90 ROC-AUC, 0.71 PR-AUC), while MHFP excels at modeling the antitumor dataset (0.89 ROC-AUC, 0.82 PR-AUC). To further inspect the classification behavior of each fingerprint, we visualized their performance in terms of precision, recall and specificity scatter plots (Fig. 4a and b), with contour lines indicating F1 score and balanced accuracy respectively. From these plots, we can conclude that MAP4, MHFP and LSTAR tend to have less false positives, while PubChem, MACCS and ESTATE generate less false negatives. Substructure fingerprints also rank particularly highly in terms of balanced accuracy (Fig. 4b), achieving a good balance of recall and specificity. When considering the post-hoc pairwise comparison tests, the situation differs from metric to metric (Additional file 1: Figure S5). Most fingerprints have statistically significant differences when considering precision, recall and specificity, while they are more comparable in terms of MCC, ROC-AUC and PR-AUC. This indicates that the false positive and true positive rate of RF models is significantly affected by the choice of molecular encoding, while the overall classification performance is less influenced.

When considering DNNs, ASP achieves the best MCC (0.562), ROC-AUC (0.8787) and PR-AUC (0.713), as shown in Additional file 1: Table S8. LSTAR is also a promising alternative, ranking first for anti-inflammatory activity modeling (0.96 ROC-AUC, 0.74 MCC) and achieving the highest precision in 3/12 datasets (Additional file 1: Table S10). One interesting difference between DNN and RF is the change in behavior of substructure-based fingerprints: while they generally lead to high recall for RF, they have more diverse performance when using DNNs. For example, PubChem here scores highly in precision, while ESTATE maintains high recall instead (Fig. 4c and d). One notable similarity between RF and DNN is that both have good performance with the MHFP fingerprint (Additional file 1: Figure S6). Given that its bit values are categorical, the expectation would be that this fingerprint would be a poor encoding choice for QSAR modeling with DNNs, since they generally assume feature cardinality. In light of these results, it is likely that the performance could be increased even further with additional preprocessing, e.g. one-hot encoding of categorical bits. Finally, when considering the post-hoc statistical tests, all methods are equal in terms of recall, while there are many significant differences in PR-AUC compared to RF (Additional file 1: Figure S6).

## Conclusions

Natural products are a promising class of compounds for drug discovery which is steadily becoming a crucial focus for biomedical research, thanks to their structural diversity, potency and selectivity in biological pathways. However, the best practices for molecular featurization of natural products is still an open question, given how different they are from typical drug-like molecules, thus limiting their use in cheminformatics applications.

Our analysis of molecular fingerprints in the natural product chemical space shows that algorithms belonging to the same category tend to be highly correlated, but they strongly diverge in terms of classification performance, pairwise similarities and chemical space representation when comparing them across categories. This finding suggests that when choosing which encoding to use for cheminformatics applications, it is beneficial to sample multiple fingerprints belonging to different classes to maximize diversity.

Concerning bioactivity prediction, our results show that the choice of molecular fingerprint has a significant impact on the classification performance across datasets (Additional file 1: Table S11). While ECFP has been the de-facto standard fingerprint for encoding drug-like compounds, our analysis indicates that other encodings can match or outperform them—the most promising ones being ASP, LSTAR and MHFP. Additionally, we highlight that while some approaches tend to perform better than others, no encoding significantly outperforms all others across all QSAR datasets in our study. This finding indicates that it is necessary to evaluate multiple fingerprints in order to obtain the best performance possible when constructing molecular property prediction models for the NP chemical space.

In terms of further fingerprint development, our study highlights two key findings. First, substructure-based fingerprints can be competitive with path and circular algorithms on NP modeling, even though they were developed for different types of molecules. As such, it would be interesting to specifically create substructure-based encodings for NPs, considering the most frequent motifs of NPs. The recently developed Natural Compound Molecular Fingerprints (NC-MFP) could be an interesting starting point for the investigation of substructure-based approaches for this class of compounds. [62]

Second, different graph traversal algorithms lead to substantially different fingerprints in terms of QSAR performance. As such, it would be interesting to pair new atom identifiers or fragment encoding algorithms with the most promising path and circular fingerprints. One particularly intriguing possibility would be to use data-driven approaches to process SMILES substrings obtained by e.g. LSTAR or ASP, potentially combining the robustness of expert-defined encodings with the expressiveness of learned molecular representations.

## Scientific contribution statement

This work is to our knowledge the first benchmarking study of molecular fingerprints for similarity searches and bioactivity prediction on natural products, a biologically relevant class of compounds that has seen limited cheminformatics modeling so far. Crucially, our findings indicate that Extended Connectivity Fingerprints, the most common encoding for drug-like compounds, can be outperformed by other molecular fingerprints, highlighting the importance of evaluating multiple encoding approaches and suggesting new research directions. Finally, we provide an open-source Python package to compute all molecular fingerprints investigated in this study to streamline their use in further cheminformatics applications.

### Supplementary Information

**Additional file 1: Table S1.** Number of compounds that were retained after each preprocessing step. Chemical structure validity was assessed via RDKIT and the ChEMBL structure curation package.1,2 Taxonomy validity was evaluated by checking whether the source organism information contained any predefined keywords, as done in a previous study by Capecchi et al. **Table S2.** Class distribution of each batch of the preprocessed subset of the COCONUT database used in this study. **Table S3.** Murcko scaffold diversity for each batch of the preprocessed subset of the COCONUT database used in this study. **Table S4.** P-values for the Mann Whitney tests with Benjamini-Hochberg correction between the similarity score distributions arising from the COCONUT and Drug Repurposing Hub datasets for each fingerprint. **Table S5.** Fingerprint saturation percentage for the COCONUT and Drug Repurposing Hub datasets. **Table S6.** Pearson correlation between using count or binary bits for a given fingerprint on the COCONUT and Drug Repurposing Hub datasets. P-values are calculated according to one-sample Mann Whitney tests with Benjamini-Hochberg correction. **Table S7.** Mean classification performance of each fingerprint using Random Forest across all datasets. **Table S8.** Mean classification performance of each fingerprint using a Dense Neural Network across all datasets.**Table S9.** Best performance rank counts for each fingerprint across all datasets for Random Forest. **Table S10.** Best performance rank counts for each fingerprint across all datasets for Dense Neural Networks. **Table S11.** Friedman test p-values evaluating the presence of significant differences in the performance of fingerprints across all datasets. **Figure S1.** Jaccard-Tanimoto similarity distribution for each fingerprint across all possible pairwise comparisons in the Drug Repurposing Hub dataset. Violin plots indicate the percentiles of the distribution of Jaccard-Tanimoto similarities, with the circle indicating the median similarity value.**Figure S2. **Correlation matrix of all pairwise similarities for all fingerprints evaluated in this study on the Drug Repurposing Hub dataset.**Figure S3.** Similarity search ranking overlap between fingerprints, focusing on the top 1% most similar compounds. **a** Rank overlap between fingerprints on the COCONUT dataset. **b** Difference in rank overlap between fingerprints when comparing the values obtained on the COCONUT and Drug Repurposing Hub datasets. Positive overlaps mean that a given fingerprint pair has a higher overlap on natural products than on drug-like compounds. Asterisks denote significance (α=0.05) according to a one-sample Mann Whitney U test with Benjamini Hochberg correction. Raw p-values are available on the Github repository of this article.**Figure S4.** Significance of the Random Forest performance differences between fingerprint pairs across all datasets, according to a 2-tailed Wilcoxon test with the Benjamini-Hochberg correction. Red denotes whether the difference is significant (α=0.05 ).**Figure S5. **Significance of the Dense Neural Network performance differences between fingerprint pairs across all datasets, according to a 2-tailed Wilcoxon test with the Benjamini-Hochberg correction. Red denotes whether the difference is significant (α=0.05).**Figure S6.** Performance comparison for each fingerprint depending on the classifier. The x-axis shows the mean ROC-AUC performance of a Random Forest classifier trained with a given fingerprint. The y-axis shows the mean ROC-AUC performance of a Dense Neural Network using different fingerprints as inputs.

## Data Availability

The Python package to compute all the fingerprints, as well as the classification metrics for each individual QSAR dataset and scripts necessary to reproduce the results presented in this study are available at https://github.com/dahvida/NP_Fingerprints.

## Abbreviations

QSAR: Quantitative Structure–Activity Relationship
ECFP: Extended Connectivity Fingerprint
MHFP: MinHash Fingerprint
NP: Natural product
TT: Topological Torsion fingerprint
AP: Atom Pair fingerprint
DFS: Depth First Search fingerprint
ASP: All Shortest Paths fingerprint
PH2: Pharmacological Pairs fingerprint
PH3: Pharmacological Triplets fingerprint
FCFP: Functional Class Fingerprint
KR: Klekotha-Roth fingerprint
MAP4: MinHashed Atom Pair fingerprint
MCC: Matthews Correlation Coefficient
ROC-AUC: Receiver Operating Characteristic Area Under Curve
PR-AUC: Precision Recall Area Under Curve
PCA: Principal Component Analysis
MST: Minimum Spanning Tree
UMAP: Unifor Manifold Approximation and Projection
RF: Random Forest
DNN: Dense Neural Network
