# Supplementary material for: Effectiveness of molecular fingerprints for exploring the chemical space of natural products
Source: J Cheminform. 2024 Mar 25;16:35. doi: 10.1186/s13321-024-00830-3 (PMC10964529; doi:10.1186/s13321-024-00830-3)
Supplement: Supplementary file 1 — Additional file 1: Table S1. Number of compounds that were retained after each preprocessing step. Chemical structure validity was assessed via RDKIT and the ChEMBL structure curation package.1,2 Taxonomy validity was evaluated by checking whether the source organism information contained any predefined keywords, as done in a previous study by Capecchi et al. Table S2. Class distribution of each batch of the preprocessed subset of the COCONUT database used in this study. Table S3. Murcko scaffold diversity for each batch of the preprocessed subset of the COCONUT database used in this study. Table S4. P-values for the Mann Whitney tests with Benjamini-Hochberg correction between the similarity score distributions arising from the COCONUT and Drug Repurposing Hub datasets for each fingerprint. Table S5. Fingerprint saturation percentage for the COCONUT and Drug Repurposing Hub datasets. Table S6. Pearson correlation between using count or binary bits for a given fingerprint on the COCONUT and Drug Repurposing Hub datasets. P-values are calculated according to one-sample Mann Whitney tests with Benjamini-Hochberg correction. Table S7. Mean classification performance of each fingerprint using Random Forest across all datasets. Table S8. Mean classification performance of each fingerprint using a Dense Neural Network across all datasets.Table S9. Best performance rank counts for each fingerprint across all datasets for Random Forest. Table S10. Best performance rank counts for each fingerprint across all datasets for Dense Neural Networks. Table S11. Friedman test p-values evaluating the presence of significant differences in the performance of fingerprints across all datasets. Figure S1. Jaccard-Tanimoto similarity distribution for each fingerprint across all possible pairwise comparisons in the Drug Repurposing Hub dataset. Violin plots indicate the percentiles of the distribution of Jaccard-Tanimoto similarities, with the circle indicating the median similarity value. [file 13321_2024_830_MOESM1_ESM.docx]

**Supporting Information**

Effectiveness of molecular fingerprints for exploring the chemical space of natural products

*Davide Boldini,^1^* Davide Ballabio,^2^ Viviana Consonni,^2^ Roberto Todeschini,^2^ Francesca Grisoni,^3,4^ Stephan A. Sieber^1^*

^1^ Technical University of Munich, TUM School of Natural Sciences, Department of Bioscience, Center for Functional Protein Assemblies (CPA), 85748 Garching bei München, Germany

^2^ Milano Chemometrics and QSAR Research Group, Department of Earth and Environmental Sciences, University of Milano-Bicocca, P.zza della Scienza, 1, 20126 Milano, Italy

^3^ Eindhoven University of Technology, Institute for Complex Molecular Systems and Dept. Biomedical Engineering, Eindhoven, Netherlands.

^4^ Centre for Living Technologies, Alliance TU/e, WUR, UU, UMC Utrecht, Utrecht, Netherlands.

*corresponding author: *davide.boldini@tum.de*

**Table S1** – Number of compounds that were retained after each preprocessing step. Chemical structure validity was assessed via RDKIT and the ChEMBL structure curation package.^1,2^ Taxonomy validity was evaluated by checking whether the source organism information contained any predefined keywords, as done in a previous study by Capecchi et al.^3^

| **Processing step** | **Number of compounds** |
| --- | --- |
| Raw COCONUT dataset | 400837 |
| Compounds with taxonomy annotation | 135064 |
| Compounds with defined taxonomy and valid chemical structure | 129869 |

**Table S2** – Class distribution of each batch of the preprocessed subset of the COCONUT database used in this study.

| **Batch ID** | **Plant %** | **Bacteria %** | **Fungi %** | **Animal %** | **Marine %** |
| --- | --- | --- | --- | --- | --- |
| All | 69.21 | 10.83 | 12.55 | 0.8 | 6.61 |
| 0 | 68.78 | 11.18 | 12.02 | 0.97 | 7.05 |
| 1 | 69.05 | 10.84 | 12.26 | 0.92 | 6.93 |
| 2 | 69.63 | 10.93 | 11.93 | 0.72 | 6.79 |
| 3 | 69.42 | 10.7 | 12.39 | 0.75 | 6.74 |
| 4 | 69.16 | 10.47 | 12.49 | 0.8 | 7.08 |
| 5 | 69.83 | 10.41 | 11.87 | 0.75 | 7.14 |
| 6 | 68.99 | 10.8 | 12.79 | 0.75 | 6.67 |
| 7 | 68.29 | 10.8 | 13.09 | 0.85 | 6.97 |
| 8 | 69.89 | 9.96 | 12.42 | 0.84 | 6.89 |
| 9 | 69.35 | 10.54 | 12.61 | 0.78 | 6.72 |
| 10 | 68.95 | 10.61 | 12.35 | 0.96 | 7.13 |
| 11 | 70.04 | 10.08 | 12.59 | 0.86 | 6.43 |
| 12 | 68.52 | 11.22 | 12.38 | 0.97 | 6.91 |
| 13 | 69.41 | 11.24 | 12.17 | 0.87 | 6.31 |
| 14 | 69.18 | 10.39 | 12.57 | 0.82 | 7.04 |
| 15 | 69.16 | 10.63 | 12.43 | 0.85 | 6.93 |
| 16 | 69.91 | 10.86 | 12.17 | 0.81 | 6.25 |
| 17 | 69.42 | 10.99 | 12.56 | 0.85 | 6.18 |
| 18 | 69.14 | 10.78 | 12.46 | 0.73 | 6.89 |
| 19 | 69.3 | 10.54 | 12.3 | 1.05 | 6.81 |
| 20 | 68.8 | 10.87 | 12.49 | 0.86 | 6.98 |
| 21 | 69.17 | 10.25 | 12.47 | 0.86 | 7.25 |
| 22 | 69.44 | 10.61 | 12.25 | 0.78 | 6.92 |
| 23 | 70.13 | 10.36 | 11.75 | 0.79 | 6.97 |
| 24 | 69.25 | 11.13 | 11.92 | 0.78 | 6.92 |
| 25 | 69.48 | 10.85 | 12.25 | 0.77 | 6.65 |
| 26 | 70.29 | 10.56 | 12.07 | 0.73 | 6.35 |
| 27 | 69.43 | 10.8 | 12.51 | 0.77 | 6.49 |
| 28 | 69.65 | 10.48 | 12.58 | 0.73 | 6.56 |
| 29 | 69.13 | 11.17 | 12.33 | 0.66 | 6.71 |
| 30 | 69.34 | 10.92 | 12.14 | 0.63 | 6.97 |
| 31 | 69.32 | 10.76 | 12.43 | 0.74 | 6.75 |
| 32 | 69.34 | 10.59 | 12.14 | 1.04 | 6.89 |
| 33 | 69.79 | 10.43 | 12.44 | 0.75 | 6.59 |
| 34 | 68.78 | 10.65 | 12.8 | 0.99 | 6.78 |
| 35 | 69.82 | 10.39 | 12.2 | 0.81 | 6.78 |
| 36 | 69.56 | 10.58 | 12.12 | 0.86 | 6.88 |
| 37 | 69.3 | 10.9 | 12.32 | 0.81 | 6.67 |
| 38 | 69.3 | 11.28 | 12.21 | 0.89 | 6.32 |
| 39 | 69.28 | 10.51 | 12.26 | 0.86 | 7.09 |
| 40 | 68.84 | 11.15 | 12.12 | 0.93 | 6.96 |
| 41 | 69.43 | 10.96 | 11.93 | 0.98 | 6.7 |
| 42 | 70.02 | 10.21 | 12.22 | 0.66 | 6.89 |
| 43 | 68.49 | 11.22 | 12.64 | 0.87 | 6.78 |
| 44 | 69.31 | 10.54 | 12.81 | 0.78 | 6.56 |
| 45 | 70.11 | 10.4 | 11.73 | 0.91 | 6.85 |
| 46 | 69.51 | 10.47 | 12.32 | 0.89 | 6.81 |
| 47 | 69.21 | 10.83 | 12.55 | 0.8 | 6.61 |
| 48 | 69.14 | 10.89 | 12.54 | 0.91 | 6.52 |
| 49 | 69.17 | 10.96 | 12.09 | 0.9 | 6.88 |

**Table S3** – Murcko scaffold diversity for each batch of the preprocessed subset of the COCONUT database used in this study.

| **Batch ID** | **Plant diversity %** | **Bacteria diversity %** | **Fungi diversity %** | **Animal diversity %** | **Marine diversity %** |
| --- | --- | --- | --- | --- | --- |
| All | 24.5 | 31.2 | 29.3 | 49.4 | 27.5 |
| 0 | 52.28 | 60.47 | 61.23 | 73.20 | 51.77 |
| 1 | 52.41 | 60.15 | 60.44 | 79.35 | 51.80 |
| 2 | 52.18 | 59.65 | 61.19 | 79.17 | 51.10 |
| 3 | 50.91 | 60.56 | 63.84 | 85.33 | 52.82 |
| 4 | 52.04 | 59.79 | 59.73 | 83.75 | 53.11 |
| 5 | 53.16 | 61.00 | 63.61 | 81.33 | 51.68 |
| 6 | 51.56 | 60.37 | 60.83 | 81.33 | 57.42 |
| 7 | 52.29 | 61.02 | 60.66 | 78.82 | 53.95 |
| 8 | 52.51 | 62.55 | 61.92 | 70.24 | 53.70 |
| 9 | 51.67 | 62.90 | 61.14 | 78.21 | 52.38 |
| 10 | 52.50 | 60.23 | 61.38 | 80.21 | 49.65 |
| 11 | 51.77 | 60.22 | 61.64 | 82.56 | 48.83 |
| 12 | 52.36 | 62.12 | 60.99 | 82.47 | 52.53 |
| 13 | 51.09 | 60.94 | 60.48 | 75.86 | 53.09 |
| 14 | 51.55 | 63.04 | 62.13 | 86.59 | 54.40 |
| 15 | 52.63 | 62.75 | 61.79 | 80.00 | 52.53 |
| 16 | 51.27 | 58.38 | 62.53 | 80.25 | 52.32 |
| 17 | 51.48 | 61.69 | 61.94 | 75.29 | 56.31 |
| 18 | 52.76 | 59.74 | 62.52 | 87.67 | 51.23 |
| 19 | 51.18 | 63.38 | 58.70 | 81.90 | 51.98 |
| 20 | 52.79 | 61.91 | 61.17 | 84.88 | 50.00 |
| 21 | 52.05 | 62.44 | 61.03 | 80.23 | 52.28 |
| 22 | 52.33 | 59.57 | 61.88 | 82.05 | 51.45 |
| 23 | 51.33 | 59.17 | 61.19 | 75.95 | 52.22 |
| 24 | 50.73 | 62.08 | 62.84 | 79.49 | 51.45 |
| 25 | 51.80 | 61.38 | 60.33 | 81.82 | 52.48 |
| 26 | 52.50 | 59.00 | 64.62 | 87.67 | 52.76 |
| 27 | 51.12 | 61.67 | 59.63 | 72.73 | 55.62 |
| 28 | 51.47 | 59.92 | 59.30 | 84.93 | 54.88 |
| 29 | 52.09 | 60.25 | 61.15 | 87.88 | 56.78 |
| 30 | 51.70 | 58.97 | 63.51 | 80.95 | 53.37 |
| 31 | 51.37 | 61.99 | 64.04 | 82.43 | 53.19 |
| 32 | 52.16 | 59.96 | 59.23 | 79.81 | 50.65 |
| 33 | 51.70 | 61.46 | 60.45 | 80.00 | 54.93 |
| 34 | 51.72 | 60.47 | 60.78 | 78.79 | 52.51 |
| 35 | 51.43 | 61.60 | 62.05 | 83.95 | 54.72 |
| 36 | 51.78 | 61.63 | 62.87 | 87.21 | 51.60 |
| 37 | 51.37 | 60.83 | 58.77 | 82.72 | 56.37 |
| 38 | 52.57 | 60.64 | 59.38 | 78.65 | 54.75 |
| 39 | 52.81 | 60.42 | 60.52 | 79.07 | 54.44 |
| 40 | 50.68 | 61.17 | 61.14 | 76.34 | 58.33 |
| 41 | 50.47 | 61.86 | 61.69 | 84.69 | 53.43 |
| 42 | 51.60 | 63.08 | 60.31 | 89.39 | 51.81 |
| 43 | 51.79 | 59.45 | 60.60 | 80.46 | 52.80 |
| 44 | 51.85 | 61.95 | 61.51 | 82.05 | 53.81 |
| 45 | 51.93 | 59.23 | 62.49 | 81.32 | 53.72 |
| 46 | 51.46 | 59.60 | 60.55 | 84.27 | 56.53 |
| 47 | 50.63 | 59.83 | 62.95 | 86.25 | 54.92 |
| 48 | 51.82 | 58.03 | 61.08 | 82.42 | 55.52 |
| 49 | 52.09 | 62.50 | 61.87 | 83.33 | 53.34 |


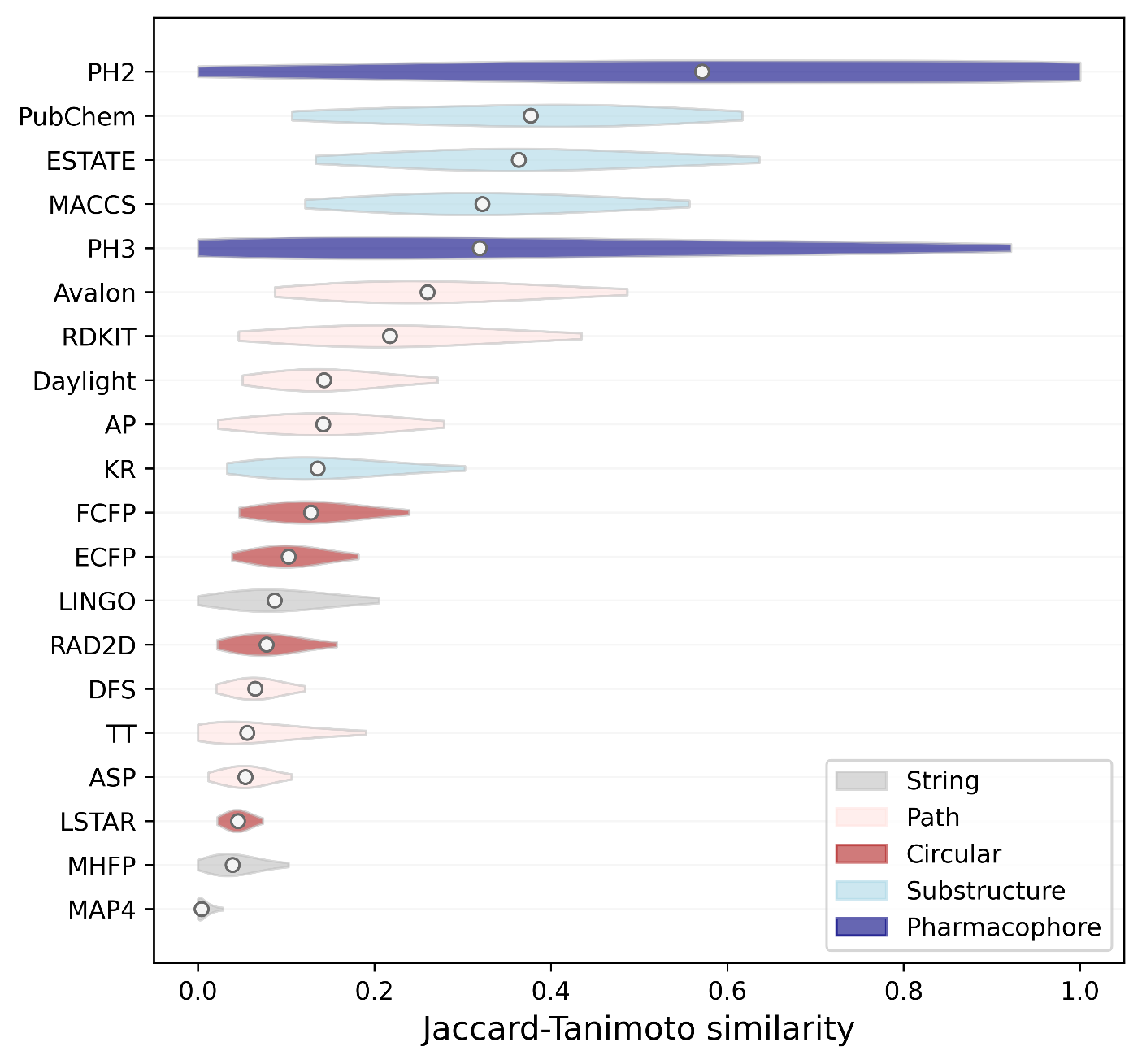


**Figure S1** – Jaccard-Tanimoto similarity distribution for each fingerprint across all possible pairwise comparisons in the Drug Repurposing Hub dataset. Violin plots indicate the percentiles of the distribution of Jaccard-Tanimoto similarities, with the circle indicating the median similarity value.

**Table S4** – P-values for the Mann Whitney tests with Benjamini-Hochberg correction between the similarity score distributions arising from the COCONUT and Drug Repurposing Hub datasets for each fingerprint.

| **Fingerprint** | **P-value** |
| --- | --- |
| AP | 0.04 |
| ASP | 0.07 |
| Avalon | 0.06 |
| Daylight | 0.07 |
| DFS | 0.06 |
| ECFP | 0.28 |
| ESTATE | 0.01 |
| FCFP | 0.20 |
| KR | 0.01 |
| LINGO | 0.06 |
| LSTAR | 0.18 |
| MACCS | 0.01 |
| MAP4 | 0.02 |
| MHFP | 0.07 |
| PH2 | 0.02 |
| PH3 | 0.01 |
| PubChem | 0.18 |
| RAD2D | 0.00 |
| RDKIT | 0.09 |
| TT | 0.44 |

**Table S5** – Fingerprint saturation percentage for the COCONUT and Drug Repurposing Hub datasets.

| **Fingerprint** | **COCONUT %** | **Drug Repurposing Hub %** |
| --- | --- | --- |
| ap | 11.0 | 7.8 |
| asp | 8.3 | 5.4 |
| avalon | 38.8 | 33.7 |
| daylight | 14.5 | 14.1 |
| dfs | 12.1 | 7.8 |
| ecfp | 5.4 | 4.3 |
| estate | 7.3 | 7.4 |
| fcfp | 3.9 | 3.4 |
| kr | 1.5 | 1.1 |
| lingo | 4.0 | 3.3 |
| lstar | 4.4 | 3.4 |
| maccs | 26.0 | 28.1 |
| map4 | N.A. | N.A. |
| mhfp | N.A. | N.A. |
| ph2 | 0.2 | 0.2 |
| ph3 | 1.4 | 0.9 |
| pubchem | 13.8 | 15.2 |
| rad2d | 1.4 | 1.3 |
| rdkit | 41.1 | 36.8 |
| tt | 2.2 | 1.5 |


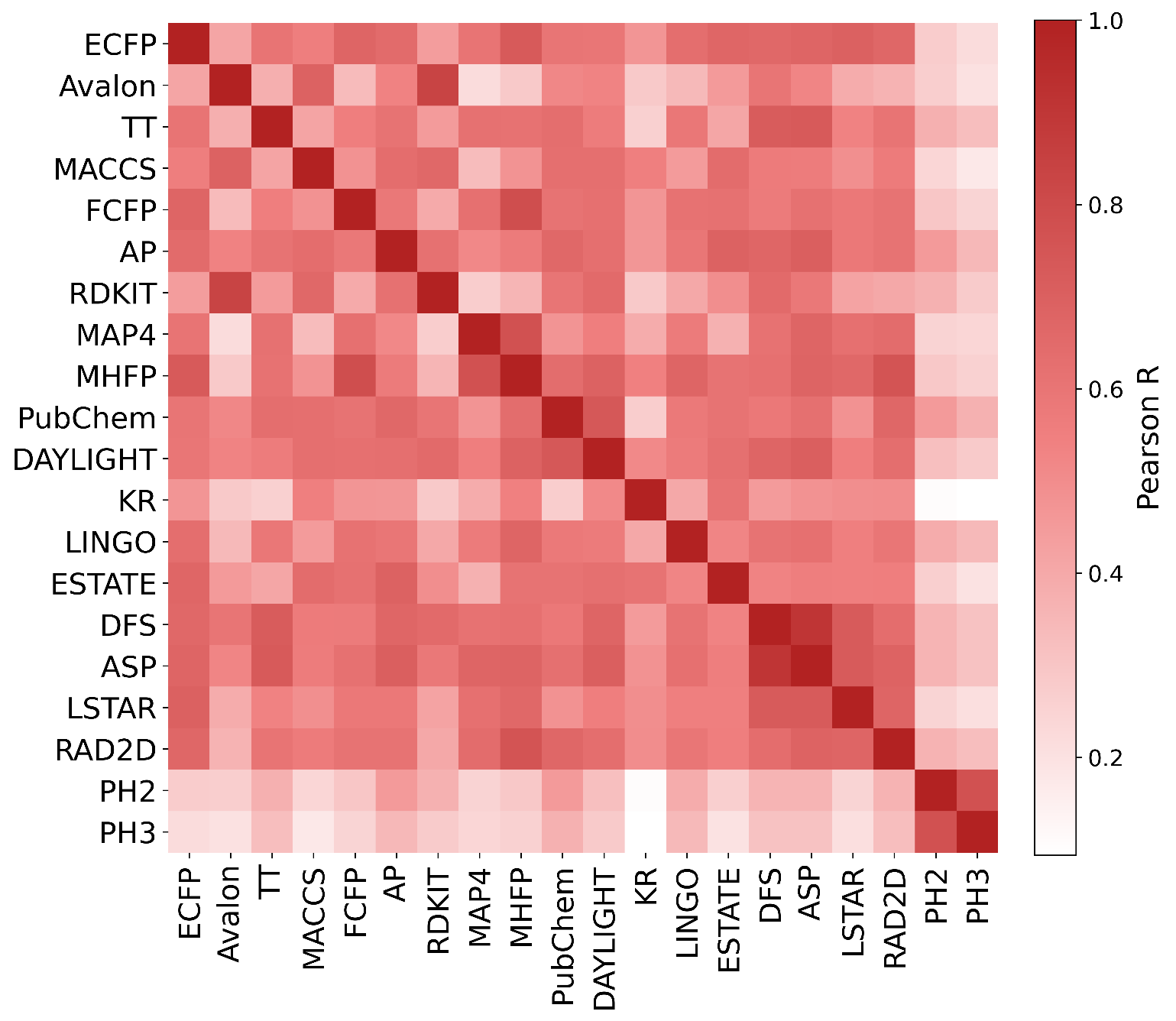


**Figure S2** - Correlation matrix of all pairwise similarities for all fingerprints evaluated in this study on the Drug Repurposing Hub dataset.


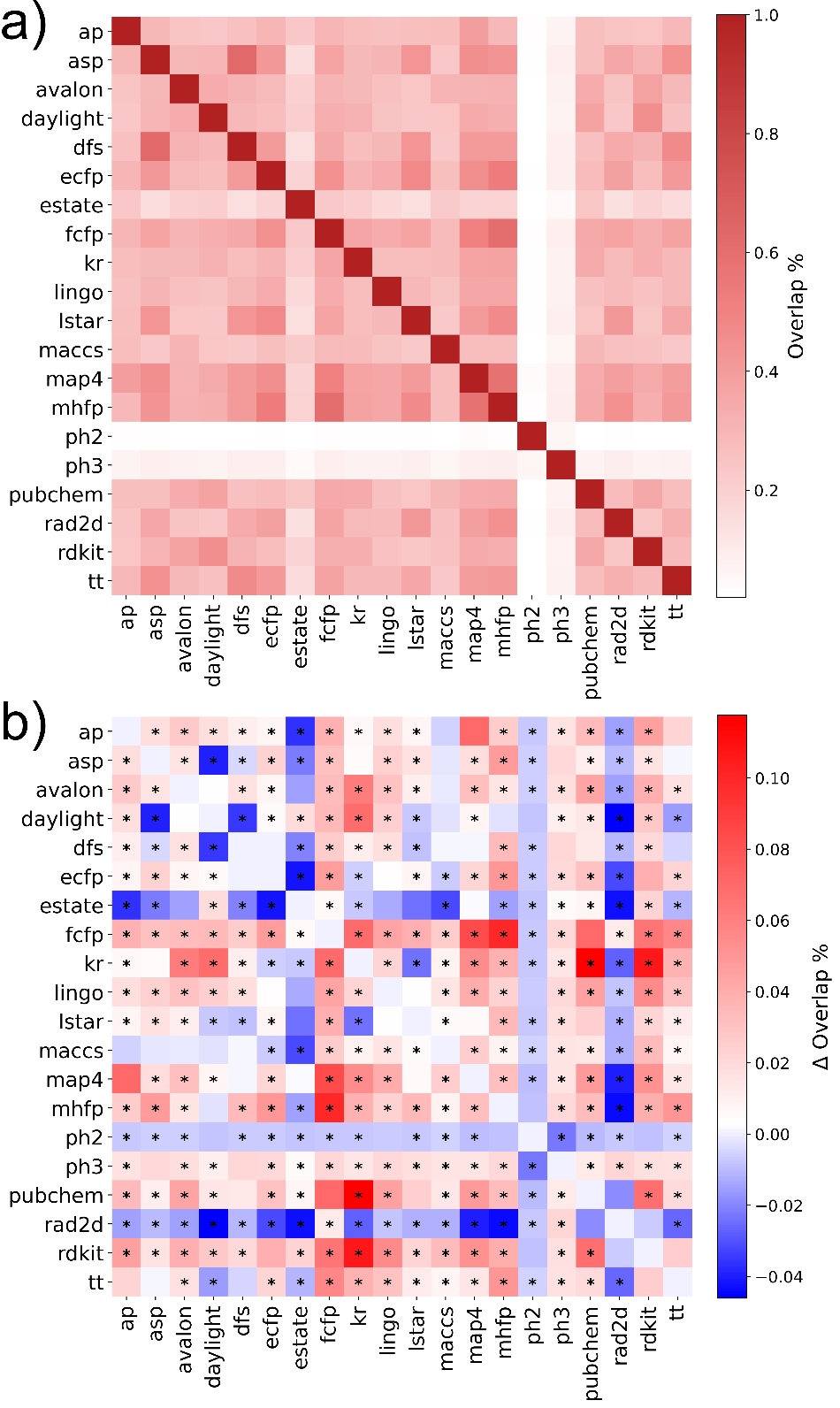


**Figure S3** – Similarity search ranking overlap between fingerprints, focusing on the top 1% most similar compounds. a) Rank overlap between fingerprints on the COCONUT dataset. b) Difference in rank overlap between fingerprints when comparing the values obtained on the COCONUT and Drug Repurposing Hub datasets. Positive overlaps mean that a given fingerprint pair has a higher overlap on natural products than on drug-like compounds. Asterisks denote significance (α=0.05) according to a one-sample Mann Whitney U test with Benjamini Hochberg correction. Raw p-values are available on the Github repository of this article.

**Table S6** – Pearson correlation between using count or binary bits for a given fingerprint on the COCONUT and Drug Repurposing Hub datasets. P-values are calculated according to one-sample Mann Whitney tests with Benjamini-Hochberg correction.

| **Fingerprint** | **COCONUT** | **Drug Repurposing Hub** | **P-value** |
| --- | --- | --- | --- |
| Atom Pair | 0.75 | 0.76 | 0.04 |
| Topological Torsion | 0.78 | 0.75 | 0.04 |
| Avalon | 0.63 | 0.66 | 0.04 |

**Table S7** – Mean classification performance of each fingerprint using Random Forest across all datasets.

| **Name** | **MCC** | **ROC-AUC** | **PR-AUC** | **Precision** | **Recall** | **Specificity** | **F1 score** | **Balanced accuracy** |
| --- | --- | --- | --- | --- | --- | --- | --- | --- |
| MAP4 | 0.443 | 0.846 | 0.597 | 0.643 | 0.430 | 0.955 | 0.515 | 0.693 |
| FCFP | 0.461 | 0.871 | 0.614 | 0.534 | 0.637 | 0.874 | 0.581 | 0.756 |
| ESTATE | 0.351 | 0.823 | 0.506 | 0.350 | 0.710 | 0.756 | 0.469 | 0.733 |
| PubChem | 0.450 | 0.873 | 0.606 | 0.492 | 0.696 | 0.826 | 0.576 | 0.761 |
| RDKIT | 0.455 | 0.866 | 0.598 | 0.549 | 0.542 | 0.918 | 0.545 | 0.730 |
| RAD2D | 0.506 | 0.893 | 0.657 | 0.599 | 0.585 | 0.921 | 0.592 | 0.753 |
| AP | 0.422 | 0.886 | 0.640 | 0.514 | 0.483 | 0.941 | 0.498 | 0.712 |
| PH2 | 0.197 | 0.672 | 0.293 | 0.248 | 0.818 | 0.450 | 0.381 | 0.634 |
| MACCS | 0.434 | 0.868 | 0.600 | 0.445 | 0.652 | 0.859 | 0.529 | 0.756 |
| MHFP | 0.443 | 0.869 | 0.669 | 0.713 | 0.383 | 0.973 | 0.498 | 0.678 |
| LSTAR | 0.423 | 0.900 | 0.662 | 0.689 | 0.425 | 0.957 | 0.526 | 0.691 |
| Daylight | 0.401 | 0.838 | 0.548 | 0.486 | 0.535 | 0.880 | 0.509 | 0.708 |
| LINGO | 0.443 | 0.862 | 0.635 | 0.584 | 0.481 | 0.935 | 0.528 | 0.708 |
| DFS | 0.472 | 0.851 | 0.622 | 0.657 | 0.476 | 0.942 | 0.552 | 0.709 |
| ASP | 0.451 | 0.863 | 0.624 | 0.598 | 0.514 | 0.910 | 0.553 | 0.712 |
| ECFP | 0.488 | 0.879 | 0.629 | 0.680 | 0.504 | 0.940 | 0.579 | 0.722 |
| Avalon | 0.419 | 0.884 | 0.622 | 0.522 | 0.491 | 0.933 | 0.506 | 0.712 |
| PH3 | 0.179 | 0.690 | 0.316 | 0.258 | 0.623 | 0.634 | 0.365 | 0.629 |
| TT | 0.474 | 0.877 | 0.647 | 0.669 | 0.480 | 0.945 | 0.559 | 0.713 |
| KR | 0.407 | 0.846 | 0.568 | 0.485 | 0.549 | 0.894 | 0.515 | 0.722 |

**Table S8** – Mean classification performance of each fingerprint using a Dense Neural Network across all datasets.

| **Name** | **MCC** | **ROC-AUC** | **PR-AUC** | **Precision** | **Recall** | **Specificity** | **F1 score** | **Balanced accuracy** |
| --- | --- | --- | --- | --- | --- | --- | --- | --- |
| MAP4 | 0.515 | 0.693 | 0.515 | 0.693 | 0.515 | 0.693 | 0.591 | 0.604 |
| FCFP | 0.581 | 0.756 | 0.581 | 0.756 | 0.581 | 0.756 | 0.657 | 0.668 |
| ESTATE | 0.469 | 0.733 | 0.469 | 0.733 | 0.469 | 0.733 | 0.572 | 0.601 |
| PubChem | 0.576 | 0.761 | 0.576 | 0.761 | 0.576 | 0.761 | 0.656 | 0.669 |
| RDKIT | 0.545 | 0.730 | 0.545 | 0.730 | 0.545 | 0.730 | 0.624 | 0.638 |
| RAD2D | 0.592 | 0.753 | 0.592 | 0.753 | 0.592 | 0.753 | 0.663 | 0.672 |
| AP | 0.498 | 0.712 | 0.498 | 0.712 | 0.498 | 0.712 | 0.586 | 0.605 |
| PH2 | 0.381 | 0.634 | 0.381 | 0.634 | 0.381 | 0.634 | 0.476 | 0.507 |
| MACCS | 0.529 | 0.756 | 0.529 | 0.756 | 0.529 | 0.756 | 0.622 | 0.642 |
| MHFP | 0.498 | 0.678 | 0.498 | 0.678 | 0.498 | 0.678 | 0.574 | 0.588 |
| LSTAR | 0.526 | 0.691 | 0.526 | 0.691 | 0.526 | 0.691 | 0.597 | 0.608 |
| Daylight | 0.509 | 0.708 | 0.509 | 0.708 | 0.509 | 0.708 | 0.592 | 0.608 |
| LINGO | 0.528 | 0.708 | 0.528 | 0.708 | 0.528 | 0.708 | 0.605 | 0.618 |
| DFS | 0.552 | 0.709 | 0.552 | 0.709 | 0.552 | 0.709 | 0.621 | 0.631 |
| ASP | 0.553 | 0.712 | 0.553 | 0.712 | 0.553 | 0.712 | 0.622 | 0.632 |
| ECFP | 0.579 | 0.722 | 0.579 | 0.722 | 0.579 | 0.722 | 0.643 | 0.650 |
| Avalon | 0.506 | 0.712 | 0.506 | 0.712 | 0.506 | 0.712 | 0.592 | 0.609 |
| PH3 | 0.365 | 0.629 | 0.365 | 0.629 | 0.365 | 0.629 | 0.462 | 0.497 |
| TT | 0.559 | 0.713 | 0.559 | 0.713 | 0.559 | 0.713 | 0.626 | 0.636 |
| KR | 0.515 | 0.722 | 0.515 | 0.722 | 0.515 | 0.722 | 0.601 | 0.618 |

**Table S9** – Best performance rank counts for each fingerprint across all datasets for Random Forest.

| **Name** | **MCC** | **ROC-AUC** | **PR-AUC** | **Precision** | **Recall** | **Specificity** |
| --- | --- | --- | --- | --- | --- | --- |
| MAP4 | 1 | 0 | 1 | 2 | 0 | 2 |
| FCFP | 2 | 0 | 0 | 0 | 1 | 0 |
| ESTATE | 0 | 0 | 0 | 0 | 2 | 0 |
| PubChem | 1 | 1 | 0 | 0 | 3 | 0 |
| RDKIT | 1 | 0 | 0 | 0 | 1 | 0 |
| RAD2D | 2 | 3 | 2 | 0 | 0 | 0 |
| AP | 0 | 1 | 0 | 0 | 0 | 1 |
| PH2 | 0 | 0 | 0 | 0 | 3 | 0 |
| MACCS | 1 | 1 | 1 | 0 | 1 | 0 |
| MHFP | 0 | 1 | 2 | 1 | 0 | 1 |
| LSTAR | 0 | 1 | 1 | 5 | 0 | 6 |
| Daylight | 0 | 0 | 0 | 0 | 0 | 0 |
| LINGO | 0 | 0 | 1 | 1 | 0 | 1 |
| DFS | 2 | 0 | 1 | 0 | 0 | 0 |
| ASP | 0 | 3 | 1 | 0 | 0 | 0 |
| ECFP | 1 | 0 | 1 | 3 | 0 | 1 |
| Avalon | 0 | 0 | 0 | 0 | 0 | 0 |
| PH3 | 0 | 0 | 0 | 0 | 1 | 0 |
| TT | 1 | 1 | 1 | 0 | 0 | 0 |
| KR | 0 | 0 | 0 | 0 | 0 | 0 |

**Table S10** – Best performance rank counts for each fingerprint across all datasets for Dense Neural Networks.

| **Name** | **MCC** | **ROC-AUC** | **PR-AUC** | **Precision** | **Recall** | **Specificity** |
| --- | --- | --- | --- | --- | --- | --- |
| MAP4 | 0 | 0 | 0 | 1 | 0 | 2 |
| FCFP | 0 | 0 | 0 | 0 | 2 | 0 |
| ESTATE | 0 | 0 | 0 | 0 | 0 | 0 |
| PubChem | 0 | 1 | 1 | 0 | 0 | 0 |
| RDKIT | 1 | 0 | 0 | 1 | 0 | 1 |
| RAD2D | 0 | 1 | 2 | 1 | 0 | 1 |
| AP | 1 | 2 | 1 | 0 | 0 | 1 |
| PH2 | 0 | 0 | 0 | 0 | 5 | 0 |
| MACCS | 2 | 1 | 1 | 1 | 0 | 0 |
| MHFP | 0 | 1 | 2 | 1 | 2 | 1 |
| LSTAR | 2 | 1 | 0 | 3 | 0 | 2 |
| Daylight | 0 | 0 | 0 | 0 | 1 | 0 |
| LINGO | 0 | 0 | 0 | 0 | 0 | 0 |
| DFS | 0 | 0 | 0 | 1 | 0 | 1 |
| ASP | 2 | 2 | 1 | 2 | 1 | 2 |
| ECFP | 1 | 1 | 3 | 1 | 0 | 1 |
| Avalon | 0 | 0 | 0 | 0 | 0 | 0 |
| PH3 | 0 | 1 | 0 | 0 | 1 | 0 |
| TT | 1 | 0 | 0 | 0 | 0 | 0 |
| KR | 2 | 1 | 1 | 0 | 0 | 0 |

**Table S11** – Friedman test p-values evaluating the presence of significant differences in the performance of fingerprints across all datasets.

| **Metric** | **Random Forest** | **Dense Neural Network** |
| --- | --- | --- |
| MCC | 1.76E-06 | 3.39E-10 |
| ROC-AUC | 8.36E-10 | 1.54E-08 |
| PR-AUC | 1.35E-11 | 1.04E-14 |
| Precision | 9.82E-15 | 9.91E-11 |
| Recall | 2.10E-11 | 0.009204 |
| Specificity | 3.14E-17 | 1.61E-10 |


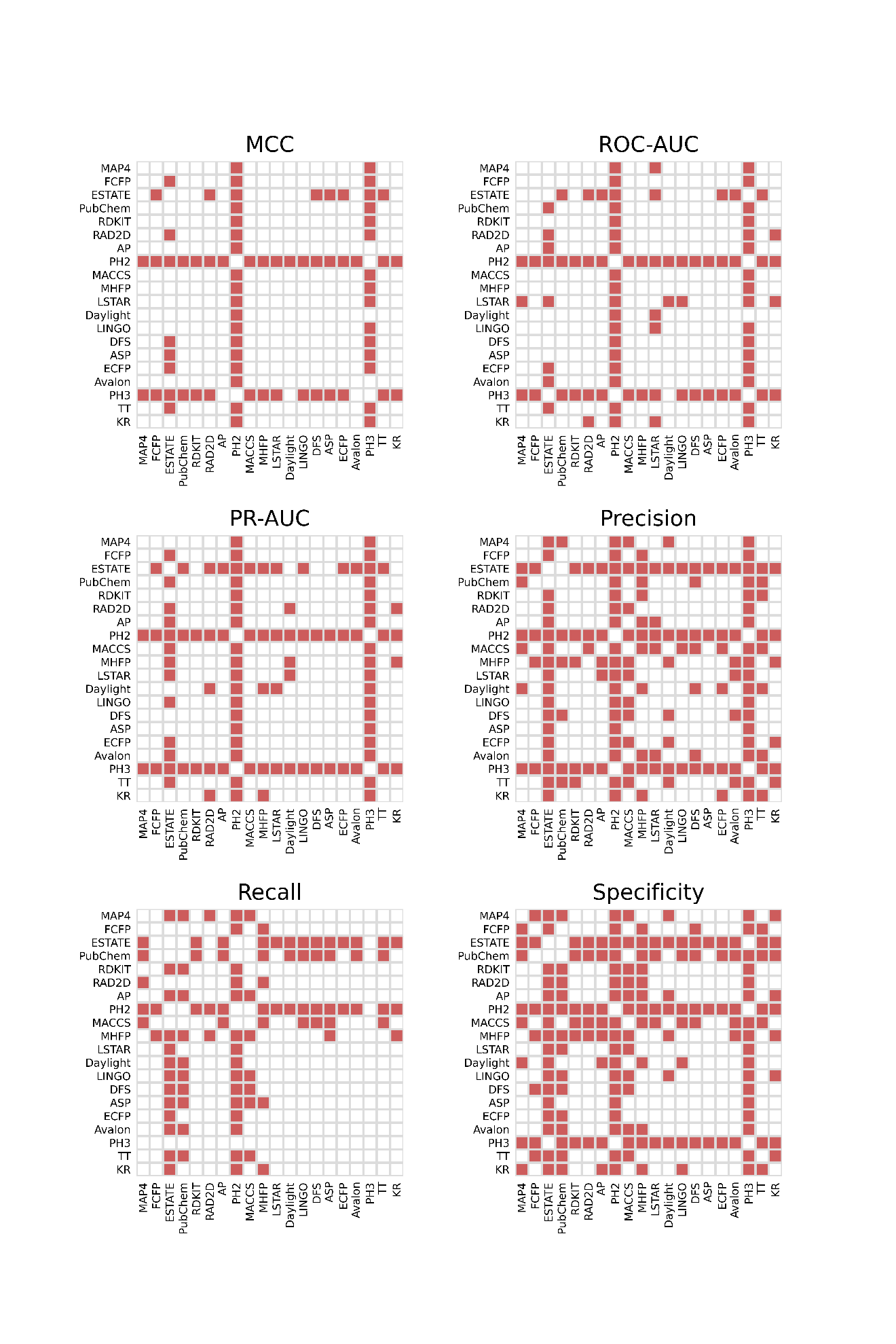


**Figure S4** – Significance of the Random Forest performance differences between fingerprint pairs across all datasets, according to a 2-tailed Wilcoxon test with the Benjamini-Hochberg correction. Red denotes whether the difference is significant (α=0.05).


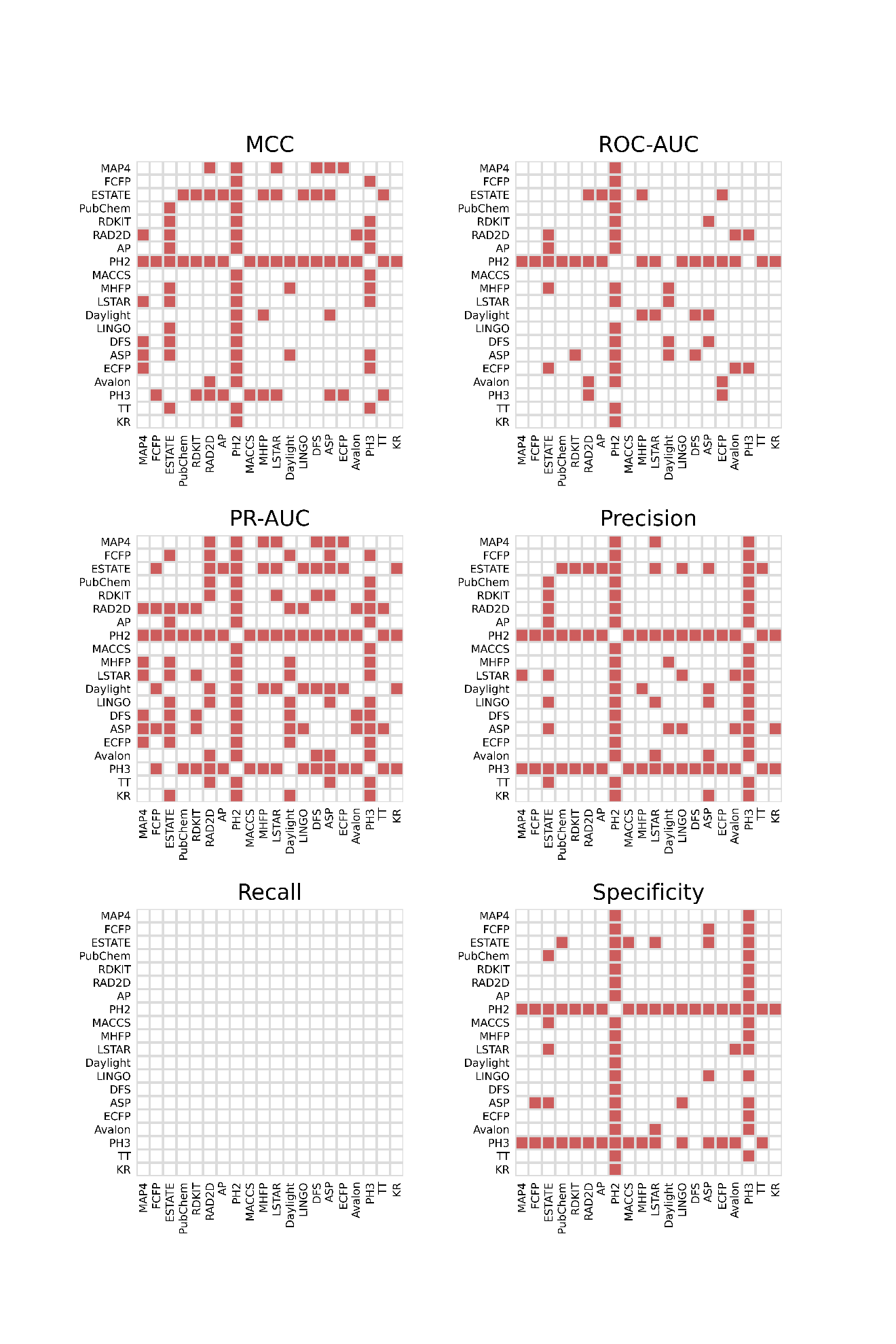


**Figure S5** – Significance of the Dense Neural Network performance differences between fingerprint pairs across all datasets, according to a 2-tailed Wilcoxon test with the Benjamini-Hochberg correction. Red denotes whether the difference is significant (α=0.05).


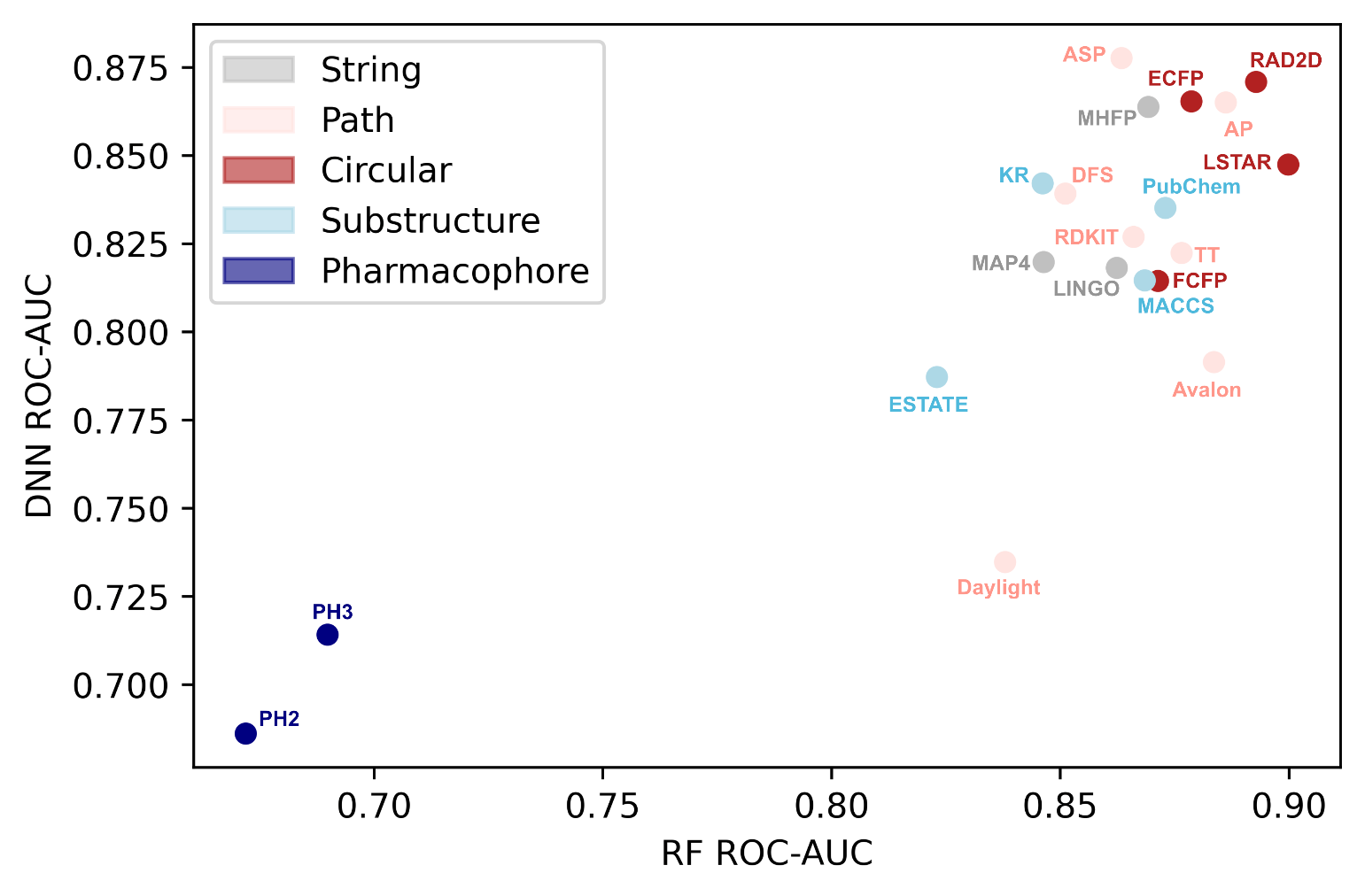


**Figure S6** – Performance comparison for each fingerprint depending on the classifier. The x-axis shows the mean ROC-AUC performance of a Random Forest classifier trained with a given fingerprint. The y-axis shows the mean ROC-AUC performance of a Dense Neural Network using different fingerprints as inputs.

**References**

(1) RDKit. https://www.rdkit.org/ (accessed 2021-05-09).

(2) Bento, A. P.; Hersey, A.; Félix, E.; Landrum, G.; Gaulton, A.; Atkinson, F.; Bellis, L. J.; De Veij, M.; Leach, A. R. An Open Source Chemical Structure Curation Pipeline Using RDKit. *J. Cheminformatics* **2020**, *12* (1), 51. https://doi.org/10.1186/s13321-020-00456-1.

(3) Capecchi, A.; Reymond, J.-L. Classifying Natural Products from Plants, Fungi or Bacteria Using the COCONUT Database and Machine Learning. *J. Cheminformatics* **2021**, *13* (1), 82. https://doi.org/10.1186/s13321-021-00559-3.
